# The phylogenetic relationship and demographic history of rhesus macaques (*Macaca mulatta*) in subtropical and temperate regions, China

**DOI:** 10.1002/ece3.11429

**Published:** 2024-05-20

**Authors:** Yanyan Zhou, Jundong Tian, Mengya Han, Jiqi Lu

**Affiliations:** ^1^ School of Life Sciences Zhengzhou University Zhengzhou China; ^2^ Institute of Biodiversity and Ecology Zhengzhou University Zhengzhou China

**Keywords:** demographic history, phylogenetic relationship, Pleistocene glaciation, Refugia, rhesus macaque (*Macaca mulatta*)

## Abstract

Pleistocene climatic oscillations exerted significant influences on the genetic structure and demography of rhesus macaque (*Macaca mulatta*) in eastern China. However, the evolutionary history of rhesus macaques in subtropical and temperate China remained unclear and/or controversial. Herein, we analyzed the autosomes, mitochondrial genomes, and Y‐chromosomes from 84 individuals of Chinese rhesus macaque. The results revealed that (1) all individuals were clustered into pan‐west and pan‐east genetic groups, which exhibited Shaanxi Province as the northernmost region of western dispersal route of rhesus macaques in China; (2) in subtropical and temperate China, rhesus macaques were divided into four lineages (TH, DB, HS, and QL), and their divergence times corresponded to the Penultimate Glaciation (300–130 kya) and Last Glaciation (70–10 kya), respectively; (3) the individuals from Mt. Taihangshan (TH) are closely related to individuals from Mt. Dabashan (DB) in the autosomal tree, rather than individuals from Mt. Huangshan (HS) as indicated by the mitogenome tree, which supports the hypothesis that the ancestral rhesus macaques radiated into Mt. Taihangshan from Mt. Huangshan via Mt. Dabashan; and (4) the demographic scenario of the four lineages showed the ancestral rhesus macaques bottleneck and expansion corresponding to the suitable habitat reduction and expansion, which confirmed they had experienced northward recolonization and southward retreat events from Mt. Huangshan area via Northern China Plain to Northernmost China along with Pleistocene glacial cycles. This study provides a new insight into understanding how Pleistocene glaciation has influenced faunal diversity in subtropical and temperate China, especially for those exhibiting differential patterns of sex dispersal.

## Graphical abstract

The multiple genomic markers including autosomes, mitochondrial genomes, and Y‐chromosomes from 84 individuals of Chinese rhesus macaque revealed that all individuals were clustered into pan‐west and pan‐east genetic groups, which exhibited Shaanxi Province as the northernmost region of western dispersal route of rhesus macaques in China. In temperate and adjacent regions of China, rhesus macaques were divided into four lineages (TH, DB, HS, and QL), and their divergence times corresponded to the Penultimate Glaciation (300–130 kya) and Last Glaciation (70–10 kya), respectively. The individuals from Mt. Taihangshan (TH) are closely related to individuals from Mt. Dabashan (DB) in the autosomal tree, rather than individuals from Mt. Huangshan (HS) as indicated by the mitogenome tree, which supports the hypothesis that the ancestral rhesus macaques radiated into Mt. Taihangshan from Mt. Huangshan via Mt. Dabashan.

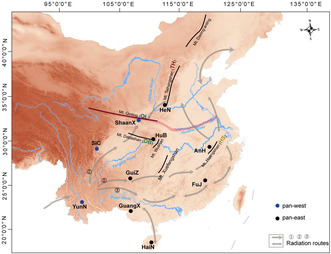

## 1 INTRODUCTION

The periodic alternation of cold and warm cycles during the Pleistocene has caused climate fluctuations and frequent shifts in vegetation (Hofreiter & Stewart, 2009; Kirschner et al., 2023; Ruiz‐Garcia et al., 2021), which has shaped the population genetic structure and demographic history of many species (He et al., 2018; Hewitt, 2004; Seersholm et al., 2020; Shepard & Burbrink, 2009; Wan et al., 2022). Published studies have investigated multiple genomic markers with different modes of inheritance and dispersal patterns to trace the complete evolutionary history of some species (Hu, Hao, et al., 2020; Hu, Thapa, et al., 2020; Kuang et al., 2019; van der Valk et al., 2020; Yang et al., 2022). There are a number of conflicting patterns of phylogeny between mitochondrial and nuclear genetic markers, which has been shown to relate to changes in the geography and climate of given species has experienced (Qian et al., 2023; Teng et al., 2017; Zhang et al., 2023).

**FIGURE 1:**
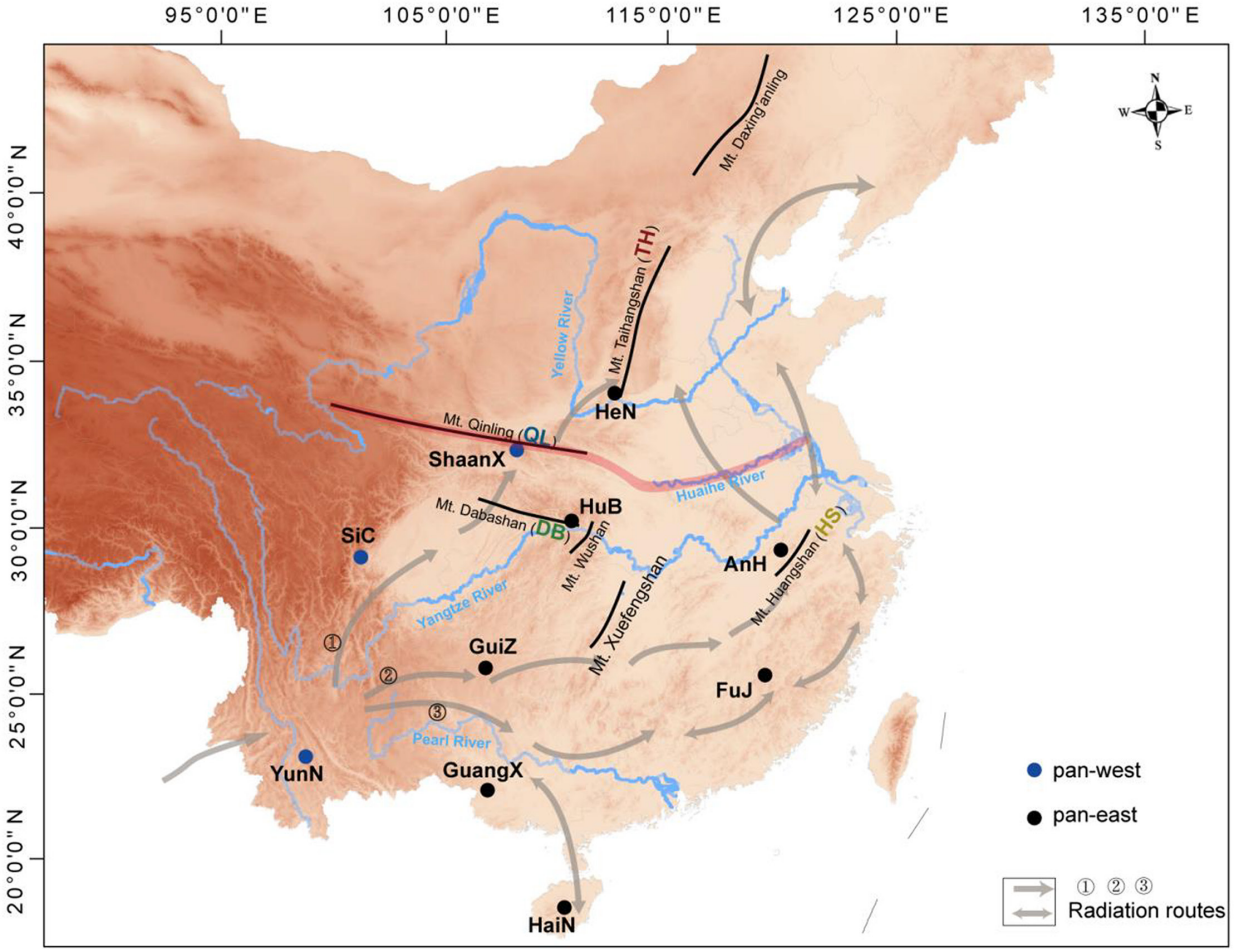
The western and eastern historical dispersal routes of Chinese rhesus macaques proposed in previous studies. The dots represented the sampling sites of Chinese rhesus macaques. The bi‐directional arrows represented rhesus macaques dispersal route of northward recolonization and southward retreat along with the Pleistocene glacial cycles. The western route (①) included individuals from western China (i.e., Yunnan = YunN, Sichuan = SiC, and Shaanxi = ShaanX Provinces), and the eastern route (② and ③) included individuals from eastern China (i.e., Guizhou = GuiZ, Guangxi = GuangX, Hainan = HaiN, Fujian = FuJ, Anhui = AnH, Hubei = HuB, and Henan = HeN Provinces). The black bold lines represented the mountains (Mts. Qinling, Dabashan, Huangshan, and Taihangshan) in subtropical and temperate China and the boundary line of the third and second steps of China's terrain (Mts. Daxing'anling, Taihangshan, Wushan, and Xuefengshan). The blue lines represented the rivers in China (Pearl, Yangtze, Yellow, and Huaihe Rivers). The red bold line represented the Qinling‐Huaihe line.

Recent researches have mainly focused on exploring mountain species (Tang et al., 2018; Yang et al., 2022; Yi et al., 2020) and indicated that intraspecific population genetic variation is linked to disparate environmental factors and geographic features (Khanal et al., 2018; Yi et al., 2020). The high mountains have been shown to act as dispersing corridors and glacial refugia for some species during the Pleistocene (Wang et al., 2013; Ye et al., 2021; Yi et al., 2020). Qinling‐Huaihe line (32°N~34°N, Figure 1) serves as the boundary between subtropical and temperate regions of China (Qin et al., 2023), and there has complex geography on the two sides of the boundary, such as high mountains (Mts. Qinling, Dabashan, Huangshan, and Taihangshan), hilly regions, and plains (Jiang & Yang, 2009). For example, the Mts. Qinling‐Dabashan extend nearly 2500 km in central China, which forms an important geographical and climatic barrier between the southern subtropical and northern temperate regions of China (Dong et al., 2022; Huang et al., 2017; Li et al., 2022). There also exist a number of micro‐refugia in the Jiangnan Hilly Region located in subtropical China, especially in Mt. Huangshan has a heterogeneous topography with mountains of elevation lower than 2000 m, which was able to maintain stable climatic conditions for some species during the Pleistocene (Wang et al., 2018; Yi et al., 2020; Zheng et al., 2016). Furthermore, Mt. Taihangshan has a northeast‐southwest trend and the southern part is a nearly east‐to‐west trend in temperate China and locates on the eastern edge of China's secondary terrain ladder and represents a natural barrier for the North China Plain (Li & Tan, 2018). Therefore, these topographic features in subtropical and temperate China play a key role in the genetic structure variation within species.

Rhesus macaque (*Macaca mulatta*) is a widely distributed primate species (Fooden, 1982; Hernandez et al., 2007) and originated in or near the northern part of the Indochinese peninsula around 2.31 million years ago (Wu et al., 2013). They entered China through the southeast corner of the Qinghai‐Tibet Plateau and then rapidly distributed from tropical rainforest to temperate snow mountains (Jiang et al., 1991; Lu, 2020; Zhang et al., 2022; Zhang & Shi, 1993). There is now a general consensus that rhesus macaques radiated into the most of China along the western and eastern routes (Liu et al., 2018; Wu et al., 2013; Zhang & Shi, 1993; Zhang et al., 2022; Zhou et al., 2024). Currently, the northernmost wild population of rhesus macaques (*M. m. tcheliensis*), with unique genetic variation (Liu et al., 2018), lower genetic diversity (Zhou et al., 2023), and the highest genetic vulnerability in the future (Wu et al., 2023), currently only distributed in southern Mt. Taihangshan area of temperate China (Lu, 2020). It has been demonstrated that understanding the evolutionary history of given species is critical for implementing efficient management conservation strategies (Hu, Hao, et al., 2020; Hu, Thapa, et al., 2020; Kuang et al., 2019). However, it has remained elusive and/or controversial how rhesus macaque initially entered into temperate China (Mt. Taihangshan area).

Previous studies based on different datasets have proposed two different dispersal routes to explain the origin of the modern population of rhesus macaque in temperate China (Figure 1). The results from mitochondrial DNA restriction enzyme (Zhang & Shi, 1993) and fossil locations/literature records (Zhang et al., 2022) indicated that rhesus macaques moved along western route (①) entering the Sichuan Basin (Sichuan Province), thence moved northwestward (Shaanxi Province) to temperate China areas. Whereas the results from mitochondrial DNA sequences and microsatellite data (Wu et al., 2013), and whole genome data (Liu et al., 2018) indicated that rhesus macaques moved eastward along the Yangtze River to Anhui Province of eastern China (② and ③), then entered the temperate China areas. This contradiction strengthens the view that integrated analyses of multiple markers with different inheritance modes are crucial to obtain a complete understanding of the population history of rhesus macaques in subtropical and temperate China. Herein, we used three genomic markers including autosomes, mitochondrial genome (mitogenome), and Y‐chromosome of rhesus macaque populations to (1) reveal the comprehensive phylogenetic relationship of Chinese rhesus macaques, (2) investigate the demographic history and divergence time of rhesus macaques in subtropical and temperate China, and (3) trace the dispersal route of rhesus macaques and uncover how they radiated into temperate China (Mt. Taihangshan area).

## 2 MATERIALS AND METHODS

### 2.1 Sampling, sequencing, and variant calling

We analyzed whole genome resequencing data of 84 individuals of Chinese rhesus macaque covering the majority of their distribution, including 80 individuals obtained from Liu et al. (2018) and four individuals from our published study (Zhou et al., 2024). In addition, two whole genome resequencing datasets of *Macaca fascicularis* (Osada et al., 2015, 2021) were used as an outgroup (Table S1). The raw reads of all individuals in this study were processed using fastp (Chen et al., 2018). The clean paired‐end reads of each individual were mapped to the reference genome (Mmul_10) using BWA v0.7.17 (Li & Durbin, 2010). BAM alignment files were generated using SAMtools (Li et al., 2009). Polymerase chain reaction duplicates were removed using PICARD. Variant calling of sequence data was performed using the GATK v4.2.3 HaplotypeCaller (McKenna et al., 2010). The applied criteria to all single nucleotide polymorphisms (SNPs) using GATK VariantFiltration was “QD < 2.0 || MQ <40.0 || FS > 60.0 || SOR > 3.0 || MQRankSum < ‐12.5 || ReadPosRankSum < ‐8.0”.

### 2.2 Identification of mitogenome and Y‐chromosome sequences

We used an in‐house Perl script described by Kuang et al. (2019) to identify individual genome sequences and sex information. The mitogenome and Y‐chromosome of each individual were determined according to the consensus sequences between the mapped short reads and mitogenome sequence (GenBank ID: AY612638) and Y‐chromosome using SAMtools mpileup (Li et al., 2009). We then filtered the consensus sequences of mitogenome without the control region and annotated ~0.88 Mb of Y‐linked genes (*SRY*, *RPS4Y1*, *ZFY*, *AMELY*, *USP9Y*, *DDX3Y*, *UTY*, *NLGN4Y*, *EIF1AY*, and *CDY*) using VCFtools v0.1.12a (Danecek et al., 2011). For individuals without sex information, we compared genomic coverage on the X‐chromosome and Y‐chromosome to identify their sex (Kuang et al., 2019). Furthermore, the reliability of the results of individual sex information was confirmed by applying it to other individuals with known sex information (Table S1).

### 2.3 Phylogeny and genetic structure analyses

The neighbor‐joining (NJ) tree with pairwise genetic distances matrix model and maximum likelihood (ML) tree with the GTRGAMMA model were constructed based on autosomal neutral SNPs using PHYLIP (Retief, 1999) and RAxML v8.0 (Stamatakis, 2014), respectively. In addition, a coalescent‐based species tree from a set of the successfully inferred NJ and ML autosomal trees was implemented in ASTRAL (Mirarab & Warnow, 2015). The mitogenome sequences were aligned using MAFFT v7.3 (Katoh & Standley, 2013) for phylogenetic construction. The best‐fit nucleotide substitution model was selected using ModelFinder (Kalyaanamoorthy et al., 2017). Bayesian approach with two parallel Markov chain Monte Carlo runs based on the HKY + F + I + G4 model was performed in MrBayes v3.2 (Ronquist et al., 2012) for 500,000 generations, with sampling every 1000 generations. In addition, the ML tree was inferred using IQ‐tree v1.6 (Nguyen et al., 2015) with the HKY + F + I + G4 model. The Y‐chromosome phylogenies following the BI tree with GTR + F + I + G4 model and ML tree with TVM + F + R2 model were constructed using MrBayes v3.2 and IQ‐tree v1.6, respectively. The phylogenetic trees were illustrated using Figtree v1.4 (Rambaut, 2009).

Principal components analysis (PCA) was carried out using PLINK v2.0 (Chang et al., 2015). Population genetic structure was inferred by ADMIXTURE v1.23 (Alexander et al., 2009) with the default setting. We increased the number of predefined genetic cluster (*K*) values from 2 to 8, and the best *K* was determined using cross‐validation (CV) analysis.

### 2.4 Introgression and incomplete lineage sorting analyses

Calculation of Patterson's *D*‐statistic was implemented in ANGSD using the ABBA_BABA test for introgression among a quartet of species (Soraggi et al., 2018). The *D*‐statistics were calculated based on multiple individuals per population using a weighted sum of the estimated allele frequencies for each individual without calling genotypes (Meleshko et al., 2021; Soraggi et al., 2018). The initial quartet topology was set as (((H1, H2), H3), outgroup), where H1, H2, and H3 represented populations from rhesus macaques in the species tree. A negative *D* value means that H1 is closer to H3 than H2, and a positive D value means H2 is closer to H3 than H1. The threshold of |*Z*| > 3 was used to determine the test significance.

The QuIBL analysis (Edelman et al., 2019) was employed based on internal branch length distribution to quantify the proportions of incomplete lineage sorting (ILS) and introgression contributions to topology incongruence. To carry out the QuIBL analysis, one fasta alignment of each population was generated from multiple individuals using ANGSD (Korneliussen et al., 2014). The 50‐kb non‐overlapping windows across autosomal sequences of each sample were extracted using SeqKit (Shen et al., 2016), and then all windows which contained samples with 50% of missing data were discarded. The best ML tree for each window was constructed using IQ‐TREE v.1.6.12 (Nguyen et al., 2015) with the GTRGAMMA model allowing for a proportion of invariable sites with 1000 ultrafast bootstrap replicates. The trees produced by IQ‐TREE and the species tree topology were used as input file for QuIBL to determine the outgroup in each case. For each triplet form W_X_Y with outgroup Y, a species pair as W_X was identified. After filtering with the significance determination value |dBIC| > 10, the discordant species pairs were identified and the average of the total IntroProp for each species pair was calculated.

### 2.5 Demographic history construction

The demographic history of rhesus macaques was traced using a pairwise sequentially Markovian coalescent (PSMC) model (Li & Durbin, 2011) based on autosomal SNPs, with the following set of parameters: ‐N25 ‐t15 ‐r5 ‐p “4 + 25*2 + 4 + 6”. Here, we chose a generation length (g) of 10.4 years and a mutation rate (μ) of 0.77 × 10^−8^ per site per generation (Bergeron et al., 2021). In addition, two complementary approaches were used to calculate historical demography and separation time between pairs of populations using SMC++ (Terhorst et al., 2017) and G‐PhoCS v1.2.2 (Gronau et al., 2011) based on different population inference models.

SMC++ uses a sequential Markovian Coalescent approach to infer the demographic history of separate rhesus macaque populations and pairwise divergence time between them based on the unphased samples with a mutation rate of *μ* = 0.77 × 10^−8^.

G‐PhoCS uses a full‐likelihood approach to infer the ancestral effective population sizes, divergence times, and migration rates. We randomly collected 1000 loci (1000 bp long) from the putative autosomal neutral regions and limited our datasets to three individuals within each population due to computational constraints. To evaluate different migration scenarios and split times between populations, nine demographic models were established and compared based on the autosomal phylogenetic topology. Every Markov chain was run for 3,000,000 iterations, sampling every 20 iterations, and the first 30,000 iterations were removed as burn‐in. For the best model, we randomly chose an additional three independent datasets and ran G‐PhoCS to check whether the estimated demographic parameters were stable. The convergence of each run was determined using Tracer v1.6 (Rambaut et al., 2013).

### 2.6 Ecological niche modeling

The potential historical spatial distribution of suitable environments for species can be identified by ecological niche models (ENM) using paleoclimate data (Peterson et al., 1999). The geographic coordinates of *Macaca* species in subtropical and temperate China (Hubei, Anhui, Shandong, Henan, Shaanxi, and Shanxi Provinces) were obtained from Li et al. (2020). The 19 bioclimatic variables for the Mid‐Holocene (~6.0 kya) (Fordham et al., 2017) and Last Interglaciation (LIG, ~130 kya) (Otto‐Bliesner et al., 2006) in 2.5 arc‐minutes, the Present and Last Glaciation Maximum (LGM, ~21 kya) periods in 30 arc‐secs (Brown et al., 2018) were downloaded from the WorldClim database (http://worldclim.org/). All bioclimatic variables were constrained to the provincial boundary of the study regions. Seven bioclimatic variables (*Bio*: 2, 3, 7, 10, 11, 15, and 18) were selected using ENMTools (Warren et al., 2010) after moving highly Pearson correlation coefficients (|*r*| ≥ .85) (Figure S1). MaxEnt v.3.4.1 (Phillips et al., 2006) was used to model and map the potential distribution of rhesus macaques in subtropical and temperate China during different time windows. For model evaluation purposes, the data was randomly divided into 75% as the training datasets and 25% as the validation datasets, with this procedure replicated 1000 times. The area under the curve of the receiver operating curve plot was used to evaluate the accuracy of the model. All models were post‐processed and visualized in ArcGIS 10.7 (http://www.esri.com/software/arcgis/arcgis‐for‐desktop).

## 3 RESULTS

### 3.1 Genome sequencing, SNP calling and sex identification

The 84 individuals of Chinese rhesus macaque were sequenced to an average depth of 11.85× and the reads had a mapping rate of 99.82% (Table S1). A total of 6.8 million high‐quality autosomal SNPs from 52 male and 32 female individuals were obtained for the following analyses (Table S1).

### 3.2 Phylogenetic relationship of Chinese rhesus macaques

**FIGURE 2:**
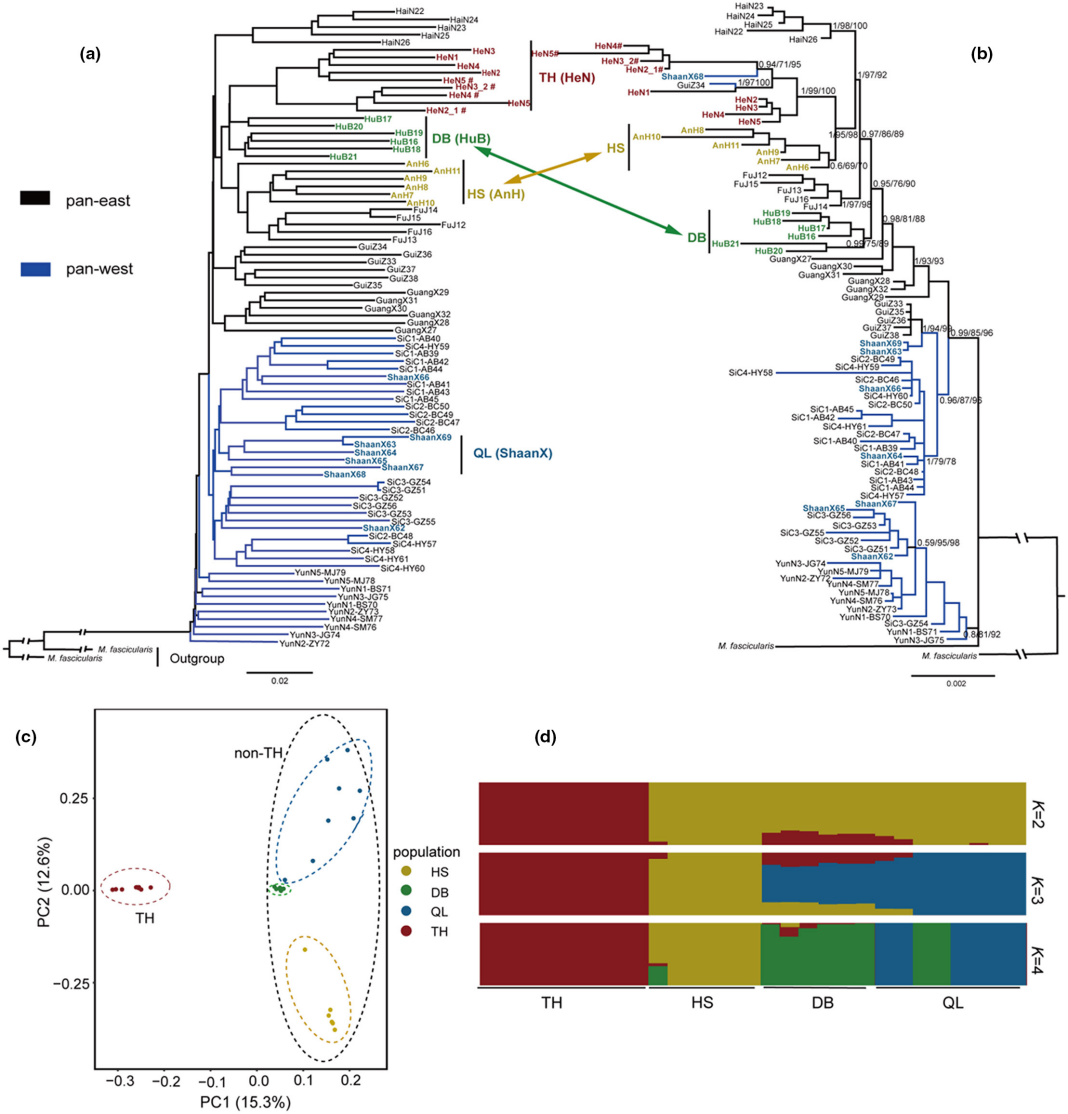
Phylogenetic relationship and population genetic analyses of Chinese rhesus macaques. (a) Phylogenetic analyses of autosomal SNPs from all individuals based on NJ, ML, and ASTRAL methods. (b) BI and ML phylogenetic trees based on mitogenomes. Node support was labeled above each node and separated by slashes: Bayesian posterior probability/Bootstrap support values. “#” indicated samples from our published study (Zhou et al., 2024). The results of 29 individuals of Chinese rhesus macaque from Mts. Qinling (QL = ShaanX), Dabashan (DB = HuB), Huangshan (HS = AnH), and Taihangshan (TH = HeN) based on autosomal SNPs (c) PCA analysis and (d) ADMIXTURE with *K* values from 2 to 4.

Phylogenetic inference (NJ and ML) based on autosomal SNPs clustered all individuals of Chinese rhesus macaque into two genetic populations (Figure 2a), which was supported by the tree inferred from the coalescent method. The autosomal trees showed that individuals from western (i.e., Yunnan, Sichuan, and Shaanxi Provinces) and eastern (i.e., Guangxi, Guizhou, Hainan, Fujian, Anhui, Hubei, and Henan Provinces) China formed pan‐west and pan‐east genetic populations, respectively (Figure 2a, Table S1).

The BI and ML trees based on the mitogenomes yielded a similar tree topology (Figure 2b). These individuals were divided into two genetic populations (pan‐west and pan‐east) with strong support (BI = 0.99, ML = 85/96), which provided additional corroborative evidence for the result of autosomal trees (Figure 2a). Notably, individuals from Shaanxi Province (QL) were nested within the pan‐west genetic population (BI = 0.96, ML = 87/96), while individuals from Anhui (HS), Hubei (DB), and Henan (TH) Provinces were clustered into the pan‐east genetic population (BI = 1, ML = 93/93). Additionally, we have noted the obvious phylogenetic discordance between the autosomal and mitogenome trees in the pan‐east genetic population (Figure 2). For instance, individuals from Henan Province (TH) are closely related to individuals from Hubei Province (DB) in the autosomal tree, rather than individuals from Anhui Province (HS) as indicated in the mitogenome tree. Beside at the population level, discordance among markers was also found at the individual level (Figure 2). For example, the individual labeled “ShaanX68” from Shaanxi Province, which is embedded in the TH lineage in the mitogenome tree, while it was clustered with the individuals from Shaanxi Province in the autosomal tree.

The phylogenies based on the Y‐chromosomes from 52 male individuals showed that all male individuals were divided into two mixed lineages (Lineage‐mix1 and Lineage‐mix2) (BI = 1, ML = 96/99) (Figure S2). The Lineage‐mix‐1 and Lineage‐mix‐2 clades consisted mostly of individuals from western and eastern China, respectively.

Our phylogenetic analyses revealed obvious discordance among trees derived from maternal, paternal, and biparental genomic markers, especially in the pan‐east genetic population. The individuals from Shaanxi Province were clustered into pan‐west genetic population in the autosome and mitogenome trees. The TH lineage is closely related to the DB lineage in the autosomal tree, rather than the HS lineage as indicated in the mitogenome tree (Figure 2). We expected that the data analysis would facilitate an understanding of how Pleistocene glaciation has influenced faunal diversity in subtropical and temperate China. Therefore, we have only focused on the evolutionary history of rhesus macaques in these areas in the following sections.

### 3.3 Genetic structure of rhesus macaques in subtropical and temperate China

The PCA results show that the TH population was genetically distinct from the non‐TH population (Figure 2c). The two populations were separated at the first eigenvector, which explained 15.3% of the total genetic variance. The second eigenvector indicated that the non‐TH population was divided into three subpopulations (QL, HS, and DB), which explained 12.6% of the total genetic variance. Moreover, there was a substantial genetic divergence between HS and DB/QL subpopulations at the second eigenvector. The ADMIXTURE analysis (Figure 2d) showed the lowest cross‐validation error when *K* = 2 (Figure S3) and supported two genetically distinct populations, which reflected a clear divergence between TH and non‐TH populations. When *K* = 4, the non‐TH individuals were divided into HS, DB, and QL subpopulations corresponding to four lineages identified in the phylogenetic and PCA analyses. In addition, the genetics of two individuals from the QL lineage were similar to the DB lineage in ADMIXTURE analysis, which provided genomic evidence of genetic similarities between the DB and QL lineages (Figure 2d).

### 3.4 Recent introgression or ILS

**FIGURE 3:**
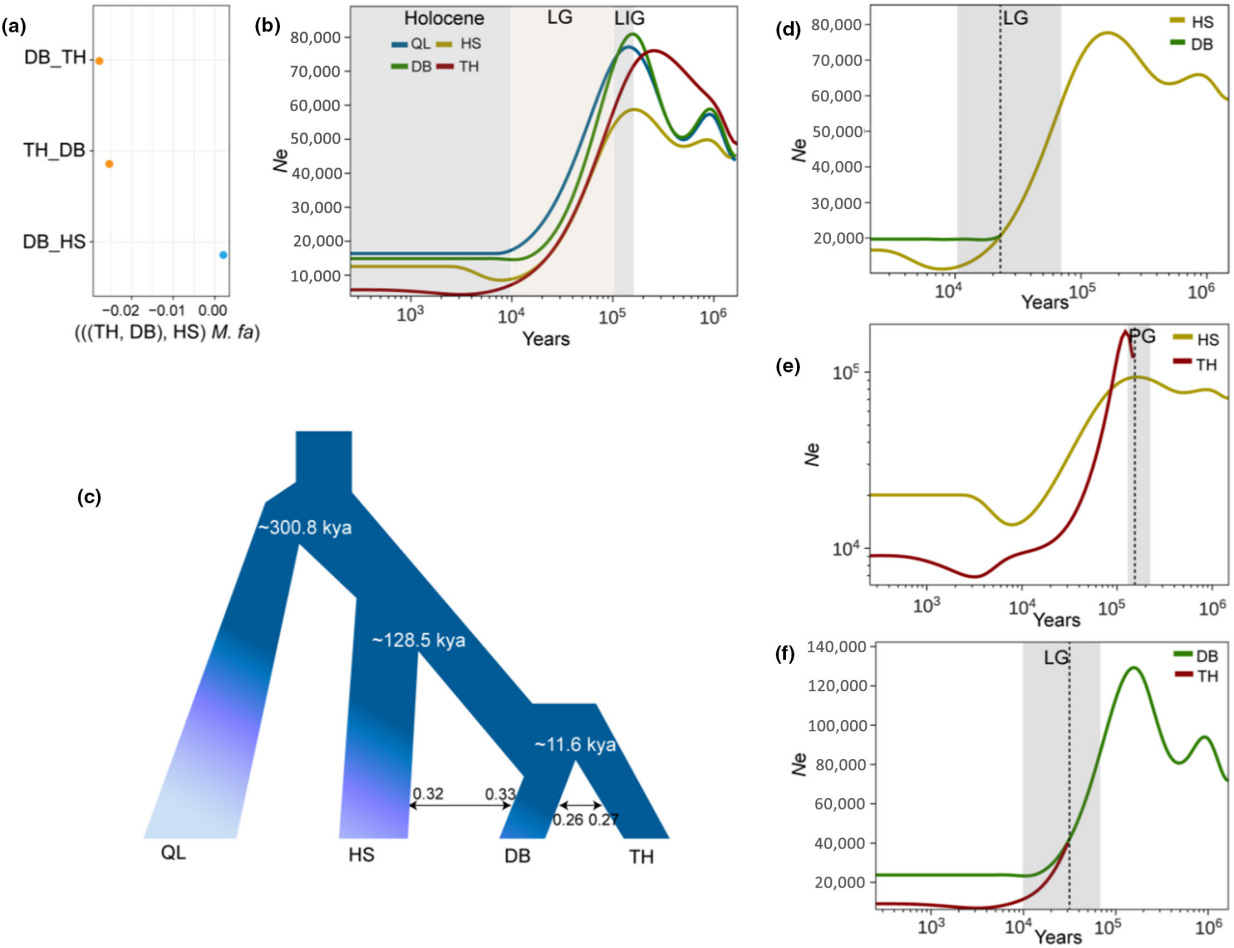
Introgression and demographic history of rhesus macaques in subtropical and temperate China. (a) The values of *D*‐statistic were calculated based on autosomal SNPs in the population pair. *M. fas*: *M. fascicularis*. (b) SMC++ inferred effective population sizes (*N*e) with respect to time (generations) for QL, DB, HS, and TH. (c) Demographic history estimated from G‐PhoCS. Arrows and numbers indicated the direction and percentage of gene flow. SMC++ inferred the divergence times of (d) HS and DB; (e) HS and TH; and (f) DB and TH. Black dotted lines indicated the separation times between different populations of Chinese rhesus macaque.

A total of three quartets conformed to the species tree topology and were detected based on their *D*‐statistic, and the quartets reached a threshold of |*Z*| > 3, which indicates a significant D value was supported by our block jackknife analysis (Table S2). In quartet topologies of the form (((TH, DB) HS) *M. fas*), negative values were not significantly different from zero in the population pair of DB_TH (Figure 3a, Table S2), which suggested that gene flow may have contributed to the observed phylogenetic discordance.

To further distinguish whether the observed phylogenetic discordance was indeed due to introgression rather than ILS, we further conducted autosomal tree set analysis in QuIBL. Among 240 triplets, only four triplets showed a significant level of ILS (Table S3). Another 236 triplets showed significant levels of introgression (ΔBIC > 10), accounting for 98.33% of the total triplet sets. Furthermore, all population pairs were identified with a threshold of totalIntroProp >0.06, which indicated that there are introgression events between populations (Table S3). Therefore, we can conclude that there is extensive gene flow between DB and TH based on the two analyses, suggesting phylogenetic conflict have been caused by gene flow between lineage pairs (DB_TH, and DB_HS), rather than ILS.

### 3.5 Demographic history

The results from PSMC analysis showed similar population size change trajectories for QL, DB, HS, and TH lineages before ~300 kya, with two apparent expansions and two severe bottlenecks in all lineages (Figure S4). The SMC++ algorithm estimated a strong population expansion (170–100 kya) and contraction (100–10 kya) of all lineages after ~300 kya (Figure 3b), which coincided with the occurrence of the Last Interglaciation (LIG, 140–120 kya) and the Last Glaciation (LG, 70–10 kya), respectively. During the Holocene (<10 kya), the population size remained stable for the QL and DB lineages, while slightly increasing and then remained stable for TH (~2 kya) and HS (5–3 kya).

### 3.6 Population divergence and correlation with climate change

We used G‐PhoCS to estimate the population divergence times and migration scenarios among the QL, DB, HS, and TH lineages. Based on a comparison of the nine models (Figure S5), Model 5 was the preferred demographic model (Akaike Information Criterion [AIC] =2674.14, Tables S4–S6), in which gene flow occurred between HS and DB (0.32–0.33) as well as between DB and TH (0.26–0.27) (Figure 3c). The divergence time estimated from the Model 5 showed that the QL clade separated from the ancestor of rhesus macaque populations ~300.8 kya (Figure 3c), which took place at the beginning of Penultimate Glaciation (PG, 300–130 kya). The HS lineage separation from the ancestor of rhesus macaques was estimated at about ~128.5 kya, occurring at the end of the PG. Then, the divergence time between DB and TH (~11.6 kya) occurred in the LG. Furthermore, SMC++ revealed that the split between HS and DB (~23 kya; Figure 3d), and between DB and TH (~31 kya; Figure 3f) occurred in the LG, and the split between HS and TH (~150 kya) occurred during the LG (Figure 3e).

**FIGURE 4:**
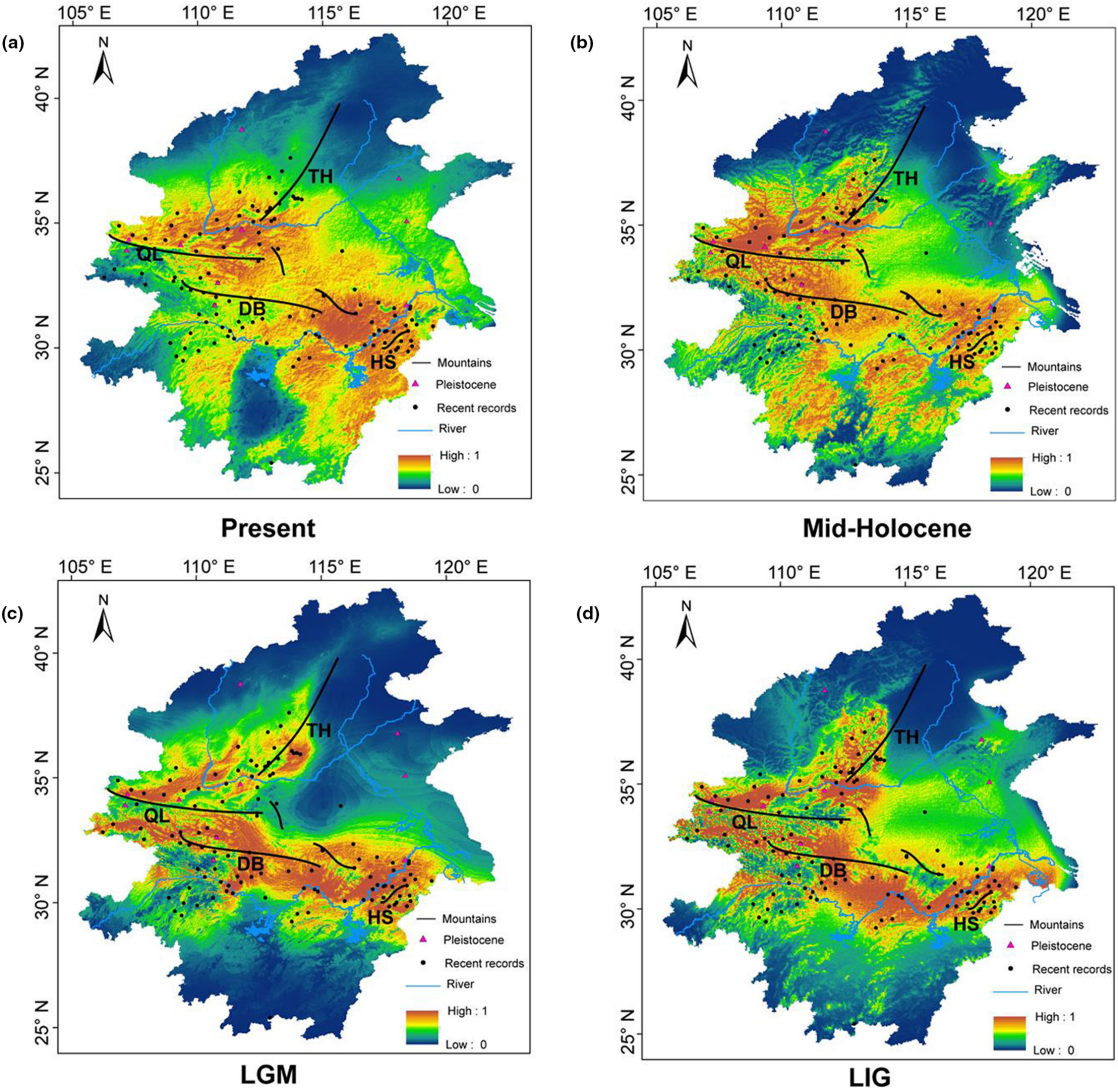
Ecological niche model projections of rhesus macaques distribution in subtropical and temperate China. Recent records (black dots) and fossil records (purple triangles) in (a) Present; (b) Mid‐Holocene (~6 kya); (c) LGM (Last Glaciation Maximum, ~21 kya); and (d) Last Interglaciation (LIG, 140–120 kya). Areas in red indicated the most suitable environmental conditions, and those in blue areas are background areas (values ranging from 0 to 1).

According to our ecological niche modeling, the suitable habitats in subtropical and temperate China for rhesus macaques likely had been affected by the Pleistocene climatic fluctuations (Figure 4). For example, the suitable habitat during the LIG was similar to the Present and the Mid‐Holocene and was more widely available than during the cold LGM. Specifically, climate change during the LGM period resulted in a heavy reduction in the suitable habitat in eastern China, such as southward contraction from the North China Plain to southern mountain areas (Figure 4c). After the LGM, the suitable habitat of Chinese rhesus macaques expanded toward warmer northern areas during the Mid‐Holocene, up to the present day (Figure 4a,b).

## 4 DISCUSSION

In this study, we used multiple genomic markers from autosomes, mitogenomes, and Y‐chromosomes from 84 individuals of Chinese rhesus macaque, and discovered that all individuals could be genetically divided into pan‐west and pan‐east populations. In temperate and adjacent subtropical areas, rhesus macaques were divided into four lineages (TH, DB, HS, and QL), and the individuals from Mt. Taihangshan (TH) are closely related to individuals from Mt. Dabashan (DB) based on autosomal tree, rather than individuals from Mt. Huangshan (HS) as indicated in our mitogenome tree. The demographic scenario of the four lineages showed the ancestral rhesus macaques bottleneck and expansion corresponding to the suitable habitat reduction and expansion, respectively.

Chinese rhesus macaques were roughly divided into pan‐west and pan‐east genetic populations based on multiple genomic markers. The boundary line of the third and second steps of China's terrain such as the north–south orientation mountains (Mts. Daxing'anling, Taihangshan, Wushan, and Xuefengshan) (Figure 1) could be the dividing line between western and eastern geographical groups of Chinese rhesus macaque. Additionally, the complex topography in western China involves high mountains, deep valleys, and large rivers (Wu & Wei, 2003), which impeded gene flow between the two genetic populations. On the other hand, in light of the male migration and female philopatry social system of rhesus macaque, we could identify the cladogenic adaptation events based on maternally inherited mtDNA (Melnick et al., 1993; Modolo et al., 2005). The positions of individuals in the Y‐chromosomal phylogenetic reflected a homogenized genetic variation facilitated by male‐mediated gene flow of rhesus macaques within the two geographic groups (Tosi et al., 2002, 2003). According to the results from multiple genomic markers analyses, individuals from western China (Sichuan = SiC and Shaanxi = ShaanX Provinces) are closely related to individuals from eastern China (Guizhou = GuiZ and Guangxi = GuangX Provinces), rather than individuals from temperate China (Henan = HeN Province). These implied that Shaanxi Province was the northernmost region of the western dispersal route of rhesus macaques in China, rather than Mt. Taihangshan in Henan Province as suggested by previous studies (Zhang et al., 2022; Zhang & Shi, 1993).

Rhesus macaques in temperate and adjacent subtropical areas were divided into four lineages (QL, DB, HS, and TH), and their genetic structure pattern corresponded to geographical distributions in Mts. Qinling, Dabashan, Huangshan, and Taihangshan areas, respectively. Moreover, the divergence times among these lineages occurred approximately during the mid‐late Pleistocene glaciation coinciding with the PG and the LG, and before that, these mountains had already risen to their present heights (Di et al., 2018; Dong et al., 2022; Shi et al., 2012; Wu et al., 2019). Glaciation appears to have more severely affected eastern than western China, which has been articled by published studies (Chu et al., 2007; Wu et al., 2013) and further confirmed by our ecological niche modeling analyses. For instance, our results from ecological niche modeling showed that these mountain valleys might have been the most stable habitats for rhesus macaques in subtropical and temperate regions during the Pleistocene glaciation periods. The east‐to‐west trend mountains, such as Mts. Qinling, Dabashan, southern Mt. Taihangshan, and Mt. Huangshan areas could have buffered regional climate variability during the Pleistocene glaciation (Hewitt, 2004) and provided glacial refugia/dispersal corridors for plants (Wang & Yan, 2014; Xu et al., 2019; Yi et al., 2020), frog species (Wang et al., 2012, 2013) as well as for rhesus macaques. Therefore, we are reasonable to deduce that the remarkable genetic differentiation among the four lineages reflects historical isolation induced by glacial refugia in these mountains during the Pleistocene glaciation. Additionally, phylogenetic analyses of the autosomes and mitogenomes showed contrasting population relationships among these lineages. For instance, the individuals from Mt. Taihangshan (TH) are closely related to individuals from Mt. Dabashan (DB) based on the autosomal tree, rather than individuals from Mt. Huangshan (HS) as indicated in the mitogenome tree. The results of the D‐statistic and QuIBL have demonstrated that the discordance was likely caused by gene flow. Extensive gene flow was detected between HS and DB as well as between DB and TH, whereas no gene flow occurred between TH and HS. In addition, the incongruence at the individual level might be attributed to male dispersal or “human introduction” because sex determination in this study provided only a small amount of information. Consequently, we deduced that rhesus macaques eastward dispersal along the second and third routes (Figure 1, ② and ③) arrived at Mt. Huangshan (Anhui Province), and then radiated into Mt. Taihangshan (Henan Province) via Mt. Dabashan (Hubei Province).

The demographic scenario of the four lineages inferred from our model simulations showed that the ancestral rhesus macaques bottleneck and expansion occurred at the suitable habitat reduction and expansion periods, respectively. For example, rhesus macaques experienced an ancestral population bottleneck when their suitable habitat contracted southwards from North China Plain to the southern mountain areas during the cold LGM, which inevitably caused a large‐scale extinction of mtDNA lineages and female “forced dispersal” (Chu et al., 2007; Wu et al., 2013). The interglacial warmer climate with suitable habitat increasing in the North China Plain had facilitated ancestral rhesus macaque population expansion and rapid northward recolonization, which could promote secondary contacts of adjacent divergent mtDNA lineages (Wu et al., 2013). Indeed, based on the fossil and literature records, Chinese rhesus macaques had a wide distribution in northern China during the Pleistocene and the Holocene (Li et al., 2020; Wen, 2018; Wen et al., 1981; Zhang et al., 2022), despite the fact that the wild rhesus macaque populations now survives only in the four isolated mountains regions in subtropical and temperate areas. This implies that the ancestral rhesus macaque population was once widespread and then became fragmented owing to human activities (arable land, roads, hunting, lumbering, etc.) in the Holocene (Li et al., 2020). These results have confirmed that rhesus macaques had experienced northward recolonization and southward retreat events from Mt. Huangshan area via the North China Plain to Northernmost China (Liaoning Province) along with Pleistocene glacial cycles.

The rhesus macaque population in subtropical and temperate China has exhibited various demographic trajectories responding to climate fluctuations and human activities. The climate changes resulted from repeated glacier and interglacial periods that occurred in the Pleistocene, which has caused several rounds of population size changes of rhesus macaque and a similar pattern in the *Budorcas taxicolor* (Yang et al., 2022) and *Rhinopithecus roxellana* (Kuang et al., 2019) in the adjacent areas of temperate China. During the Holocene, the climate might have exerted less impact on rhesus macaque populations, while increasing human activities became the main factor driving recent rhesus macaque population size changes (Zhou et al., 2023). Mts. Qinling and Dabashan have complex topography and might have suffered less impact from human activities, which resulted in stable population sizes of the QL and DB lineages. Furthermore, both the most suitable climate and habitat expansions in Mt. Huangshan contributed to a slight increase in the population size of the HS lineage 6000–4000 years ago (Xu, 1992). The cold and dry climates which have continued in the Holocene after the LG in northern China (Xu, 2001), combined with human activities (Li et al., 2020; Lu, 2020; Zhang et al., 2022; Zhou et al., 2023), have negatively affected the population size of the TH lineage.

## AUTHOR CONTRIBUTIONS

**Yanyan Zhou:** Formal analysis (equal); methodology (equal); software (equal); visualization (equal); writing – original draft (equal). **Jundong Tian:** Data curation (supporting); funding acquisition (supporting); methodology (supporting); resources (supporting). **Mengya Han:** Methodology (supporting); software (supporting). **Jiqi Lu:** Conceptualization (equal); funding acquisition (equal); project administration (equal); resources (equal); supervision (equal); writing – review and editing (equal).

## FUNDING INFORMATION

This work was financially supported by the National Natural Science Foundation of China (nos. 31672302 and 32070446) and the Cultivation Fund for Young Teachers in Natural Science Basic Research of Zhengzhou University (JC2020043029).

## CONFLICT OF INTEREST STATEMENT

The authors declare that they have no competing interests.

## Supporting information


Data S1.

## Data Availability

The genome sequences information has been showed in Table S1.
