# Supplementary material for: The phylogenetic relationship and demographic history of rhesus macaques (Macaca mulatta) in subtropical and temperate regions, China
Source: Ecol Evol. 2024 May 20;14(5):e11429. doi: 10.1002/ece3.11429 (PMC11103769; doi:10.1002/ece3.11429)
Supplement: Supplementary file 1 — Data S1. [file ECE3-14-e11429-s001.pdf]

Table S1. Sample information and sequencing data summary of the Chinese rhesus macaque and *Macaca fascicularis* individuals used in this study.

Bioproject	Run	BioSample	**a:**Sex	Depth (×)	Mapping rate (%)	**b:**Y-chromosome/ autosomes genomic coverage ratio	**b:**X-chromosome /autosomes genomic coverage ratio	Sample sites	Sampling site symbol
	THS2-1	Published study	**Female**	17.94	99.7	0.01	0.99	Henan Province: Mt. Taihangshan	HeN2-1
	THS3-2	Published study	**Male**	22.94	99.88	0.40	0.51	Henan Province: Mt. Taihangshan	HeN3-2
	THS4	Published study	Female	21.81	99.4	0.01	1.02	Henan Province: Mt. Taihangshan	HeN4
	THS5	Published study	**Female**	22.07	96.11	0.01	0.94	Henan Province: Mt. Taihangshan	HeN5
	SRR5007015<br>SRR4454029	SAMN05883675	Female	6.61	99.87	0.01	0.90	Henan Province: Mt. Taihangshan	HeN2
	SRR5009873<br>SRR4454030	SAMN05883676	Male	6.63	99.80	0.42	0.48	Henan Province: Mt. Taihangshan	HeN3
	SRR5009874<br>SRR4454031	SAMN05883677	Male	8.75	99.88	0.50	0.46	Henan Province: Mt. Taihangshan	HeN4
	SRR4454025	SAMN05883678	Male	6.01	99.87	0.46	0.45	Henan Province: Mt. Taihangshan	HeN5
	SRR4454026	SAMN05883679	Male	10.37	99.9	0.31	0.51	Henan Province: Mt. Taihangshan	HeN1
	SRR4454035	SAMN05883673	Male	5.98	99.87	0.39	0.44	Anhui Province: Mt. Huangshan	AnH6
	SRR4454028	SAMN05883674	Male	9.43	99.76	0.33	0.52	Anhui Province: Mt. Huangshan	AnH7
	SRR4454004	SAMN05883745	Female	8.45	99.7	0.00	1.07	Anhui Province: Mt. Huangshan	AnH8
	SRR4454005	SAMN05883746	Male	11.77	99.86	0.33	0.54	Anhui Province: Mt. Huangshan	AnH9
	SRR4454006	SAMN05883747	Male	10.93	99.86	0.29	0.55	Anhui Province: Mt. Huangshan	AnH10
	SRR4453998	SAMN05883748	Male	7.14	99.88	0.33	0.52	Anhui Province: Mt. Huangshan	AnH11
	SRR5019554	SAMN06016319	Female	27.04	99.83	0.01	1.02	Hubei Province: Mt. Dabashan	HuB20
	SRR4453985	SAMN05883697	Male	9.88	99.89	0.36	0.51	Hubei Province: Mt. Dabashan	HuB17
	SRR4454015	SAMN05883698	Male	8.38	99.89	0.29	0.53	Hubei Province: Mt. Dabashan	HuB18
	SRR4453996	SAMN05883699	Female	8.23	99.86	0.00	1.05	Hubei Province: Mt. Dabashan	HuB19
	SRR4454038	SAMN05883700	Female	8.86	99.84	0.01	1.09	Hubei Province: Mt. Dabashan	HuB16
	SRR5019559<br>SRR4454039	SAMN05883701	Male	41.05	99.65	0.40	0.51	Hubei Province: Mt. Dabashan	HuB21
	SRR4453972	SAMN05883730	Female	10.64	99.89	0.00	1.09	Shaanxi Province: Mt. Qinling	ShaanX62
	SRR4453971	SAMN05883731	Male	10.82	99.89	0.27	0.55	Shaanxi Province: Mt. Qinling	ShaanX63
	SRR4453970	SAMN05883732	Male	10.62	99.9	0.27	0.54	Shaanxi Province: Mt. Qinling	ShaanX64
	SRR4453969	SAMN05883733	Male	12.15	99.9	0.29	0.55	Shaanxi Province: Mt. Qinling	ShaanX65
	SRR4453968	SAMN05883734	Male	9.95	99.91	0.28	0.54	Shaanxi Province: Mt. Qinling	ShaanX66
	SRR4453967	SAMN05883735	Male	12.20	99.91	0.29	0.52	Shaanxi Province: Mt. Qinling	ShaanX67
	SRR4453966	SAMN05883736	Female	10.81	99.91	0.01	1.09	Shaanxi Province: Mt. Qinling	ShaanX68
	SRR4453965	SAMN05883737	Female	8.45	99.9	0.00	1.06	Shaanxi Province: Mt. Qinling	ShaanX69

	SRR4454022	SAMN05883681	Male	6.08	99.86	0.31	0.53	Fujian Province:Nanping County	FuJ12
	SRR4453992	SAMN05883682	Male	10.25	99.9	0.33	0.53	Fujian Province:Nanping County	FuJ13
	SRR4453993	SAMN05883683	Male	9.12	99.78	0.38	0.53	Fujian Province:Nanping County	FuJ14
	SRR4454036	SAMN05883684	Male	9.29	99.83	0.38	0.53	Fujian Province:Nanping County	FuJ15
	SRR4453990	SAMN05883685	Female	9.21	99.84	0.00	1.07	Fujian Province:Nanping County	FuJ16
	SRR4454014	SAMN05883725	Female	9.82	99.88	0.00	1.10	Guangxi Province: Mt. Longhushan	GuangX28
	SRR4454011	SAMN05883726	Male	9.87	99.87	0.26	0.55	Guangxi Province: Mt. Longhushan	GuangX29
	SRR4454012	SAMN05883727	Female	12.08	99.91	0.01	1.11	Guangxi Province: Mt. Longhushan	GuangX30
	SRR4454021	SAMN05883680	Male	5.99	99.87	0.32	0.48	Guangxi Province: Mt. Longhushan	GuangX27
	SRR4454017	SAMN05883728	Female	11.61	99.88	0.01	1.09	Guangxi Province: Mt. Longhushan	GuangX31
	SRR4454018	SAMN05883729	Female	10.25	99.91	0.00	1.07	Guangxi Province: Mt. Longhushan	GuangX32
	SRR4453963	SAMN05883739	Male	9.09	99.91	0.31	0.52	Guizhou Province: Mt. Qianlingshan	GuiZ33
	SRR4453999	SAMN05883740	Male	9.16	99.91	0.25	0.50	Guizhou Province: Mt. Qianlingshan	GuiZ34
	SRR4454000	SAMN05883741	Male	9.40	99.88	0.26	0.50	Guizhou Province: Mt. Qianlingshan	GuiZ35
	SRR4454001	SAMN05883742	Female	8.75	99.87	0.00	1.09	Guizhou Province: Mt. Qianlingshan	GuiZ36
PRJNA345528	SRR4454002	SAMN05883743	Female	8.74	99.88	0.01	1.02	Guizhou Province: Mt. Qianlingshan	GuiZ37
	SRR4454003	SAMN05883744	Male	10.55	99.9	0.28	0.53	Guizhou Province: Mt. Qianlingshan	GuiZ38
	SRR4454010<br>SRR5019558	SAMN05883721	Male	39.21	99.85	0.42	0.51	Hainan Province:Nanwan	HaiN22
	SRR4454007	SAMN05883722	Male	9.34	99.87	0.33	0.53	Hainan Province:Nanwan	HaiN23
	SRR4453964	SAMN05883738	Male	9.26	99.81	0.22	0.47	Hainan Province:Nanwan	HaiN26
	SRR4454008	SAMN05883723	Male	9.12	99.84	0.32	0.54	Hainan Province:Nanwan	HaiN24
	SRR4454013	SAMN05883724	Female	9.39	99.83	0.00	1.05	Hainan Province:Nanwan	HaiN25
	SRR4454016	SAMN05883687	Female	9.01	99.9	0.01	1.01	Yunnan Province: Mt. Baoshan	YunN1-BS70
	SRR4453989	SAMN05883688	Female	9.55	99.91	0.01	1.02	Yunnan Province: Mt. Baoshan	YunN1-BS71
	SRR5019556<br>SRR4453997	SAMN05883689	Female	40.36	99.88	0.01	0.98	Yunnan Province:Jinggu County	YunN3-JG74
	SRR4453984	SAMN05883690	Female	9.89	99.82	0.00	1.01	Yunnan Province:Jinggu County	YunN3-JG75
	SRR5019369<br>SRR4453983	SAMN05883691	Female	36.80	99.85	0.01	0.99	Yunnan Province:Mojiang County	YunN5-MJ79
	SRR4453995	SAMN05883692	Female	9.63	99.91	0.00	0.96	Yunnan Province:Mojiang County	YunN5-MJ78
	SRR4454034	SAMN05883693	Female	9.54	99.9	0.00	1.04	Yunnan Province:Simao County	YunN4-SM76
	SRR4453988	SAMN05883694	Female	9.26	99.92	0.00	1.00	Yunnan Province:Simao County	YunN4-SM77
	SRR4453987	SAMN05883695	Female	9.66	99.92	0.00	1.02	Yunnan Province:Zhenyuan County	YunN2-ZY73
	SRR5019557<br>SRR4453986	SAMN05883696	Female	38.73	99.81	0.01	0.99	Yunnan Province:Zhenyuan County	YunN2-ZY72
	SRR5019367<br>SRR4454023	SAMN05883704	Female	38.59	99.84	0.00	0.98	Sichuan Province:Hanyuan County	SiC4-HY58
	SRR4454037	SAMN05883707	Male	8.26	99.87	0.27	0.52	Sichuan Province:Hanyuan County	SiC4-HY59
	SRR4454009	SAMN05883720	Male	9.43	99.88	0.27	0.49	Sichuan Province:Hanyuan County	SiC4-HY61
	SRR4453991	SAMN05883672	Male	7.91	99.84	0.30	0.52	Sichuan Province:Hanyuan County	SiC4-HY57
	SRR4453978	SAMN05883714	Male	8.99	99.91	0.24	0.49	Sichuan Province:Hanyuan County	SiC4-HY60

	SRR4453973	SAMN05883711	Male	9.93	99.92	0.29	0.51	Sichuan Province:Aba County	SiC1-AB42
	SRR4454019	SAMN05883708	Male	8.87	99.91	0.29	0.52	Sichuan Province:Aba County	SiC1-AB41
	SRR4453977	SAMN05883715	Male	8.06	99.91	0.31	0.50	Sichuan Province:Aba County	SiC1-AB43
	SRR4453961	SAMN05883686	Female	9.39	99.83	0.01	1.06	Sichuan Province:Aba County	SiC1-AB45
	SRR4453994	SAMN05883671	Female	9.58	99.87	0.01	1.04	Sichuan Province:Aba County	SiC1-AB39
	SRR4454033	SAMN05883706	Male	9.29	99.91	0.25	0.52	Sichuan Province:Aba County	SiC1-AB40
	SRR4453982	SAMN05883718	Male	9.34	99.92	0.26	0.48	Sichuan Province:Aba County	SiC1-AB44
	SRR4454020	SAMN05883709	Male	9.35	99.83	0.30	0.54	Sichuan Province:Ganzi County	SiC3-GZ53
	SRR4454032	SAMN05883670	Male	9.18	99.86	0.27	0.53	Sichuan Province:Ganzi County	SiC3-GZ51
	SRR4453974	SAMN05883710	Male	8.65	99.9	0.26	0.46	Sichuan Province:Ganzi County	SiC3-GZ54
	SRR4453981	SAMN05883719	Male	10.99	99.88	0.25	0.48	Sichuan Province:Ganzi County	SiC3-GZ56
	SRR4454027	SAMN05883705	Male	9.39	99.92	0.28	0.49	Sichuan Province:Ganzi County	SiC3-GZ52
	SRR4453976	SAMN05883712	Male	8.42	99.81	0.25	0.53	Sichuan Province:Ganzi County	SiC3-GZ55
	SRR4453975	SAMN05883713	Male	9.96	99.86	0.25	0.49	Sichuan Province:Beichuan County	SiC2-BC48
	SRR4453980	SAMN05883716	Male	9.61	99.91	0.23	0.49	Sichuan Province:Beichuan County	SiC2-BC49
	SRR4453979	SAMN05883717	Male	9.59	99.89	0.26	0.49	Sichuan Province:Beichuan County	SiC2-BC50
	SRR4453962	SAMN05883702	Male	9.46	99.91	0.26	0.49	Sichuan Province:Beichuan County	SiC2-BC46
	SRR4454024	SAMN05883703	Male	9.74	99.84	0.27	0.49	Sichuan Province:Beichuan County	SiC2-BC47
PRJDB9555	DRR219369	SAMD00215308	Female	40.53	99.78	0.01	1.19		*M. fascicularis*
PRJEB7871	ERR710414<br>ERR749181	SAMEA3176782	Male	18.45	99.86	0.24	0.49		*M. fascicularis*

a:The sex information of the individuals with known sexual information from the sampling in the present study or previous studies reported was shown in blod. The sex information of the remaining individuals was identified by comparing genomic coverage on the X-chromosome and Y-chromosome, respectively, with genomic coverage on autosomal chromosomes. The reliability of the results was also confirmed by applying it to the individuals with known sex information, which obtained consistent results.

b:Average read-depth ratio was calculated by dividing the average read-depth in Y-chromosome and X-chromosome by the average read-depth of autosomes, respectively.

Table S2. The ABBABABA (D-stat) analysis results based on Angsd software.

The samples used in this analysis are show in Table S1.

H4 The outgroup for that line depends on the species tree.

H1, H2, andH3 are the 3 populations in the tree that are not the outgroup.

nABBA the total counts of ABBA patterns

nBABA the total counts of BABA patterns

D The test statistic: (nABBA-nBABA)/(nABBA+nBABA). A negative value means that H1 is closer to H3 than H2 is. A positive value means that H2 is closer to H3 than H1 is.

JackEst column is another estimate of the abbababa statistic that is bias corrected. This value is extremely similar to the value in the Dstat column

Z value that can be used to determine the significance of the test. As in Reich et al. an absolute value of the Z score above 3 is often used as a critical value. However, this note that this does not take into account the fact that we perform multiple tests.

H1	H2	H3	H4	D	JK-D	V(JK-D)	Z	pvalue	nABBA	nBABA	nBlocks	pair
TH	DB	HS	*M. fascicularis*	0.001871	0.001871	0.000001	1.684301	0.092123	1089312.161	1085243.224	546	DB_HS
TH	HS	DB	*M. fascicularis*	-0.025889	-0.025889	0.000001	-21.504703	0	1089312.161	1147214.193	546	TH_DB
DB	HS	TH	*M. fascicularis*	-0.027759	-0.027759	0.000001	-23.558718	0	1085243.224	1147214.193	546	DB_TH

Table S3 The QuIBL analysis results.

The samples used in this analysis are show in Table S1.

triplet : The triplet analyzed, separated by underscores.

outgroup : The outgroup for that line depends on the species tree.

C1, C2 : The time during which two lineages are sequestered for that triplet topology for a 1 distribution（a pure ILS model） and 2 distribution model（a model with a non-ILS component,

whether due to speciation and/or introgression respectively.

mixprop1, mixprop2 : The inferred mixing proportions for each distribution. mixprop2 corresponds to the non-ILS component.

BIC2Dist, BIC1Dist : BIC scores for the two models for model selection.

count : The total number of trees in that triplet topology.

discordant :Whether the topology conflicts with the species tree topology

dBIC : BIC1Dist-BIC2Dist.

totalIntroProp: Proportion of trees arising via introgression.

chr	triplet	outgroup	True outgroup	C 1	C 2	mixprop1	mixprop2	lambda2 Dist	lambda1Dist	BIC2Dist	BIC1Dist	count	discordant	dBIC	TotalIntro-Prop	pair
1	HS_TH_DB	TH	HS	0	0.87	0.75	0.25	0.00	0.00	-18749.85	-18732.65	1372.00	Ture	17.20	0.39	TH_DB
1	TH_DB_QL	DB	QL	0	0.59	0.54	0.46	0.00	0.00	-18265.28	-18195.90	1310.00	Ture	69.38	0.69	TH_DB
1	HS_TH_QL	TH	QL	0	0.59	0.53	0.47	0.00	0.00	-19045.81	-18981.83	1384.00	Ture	63.98	0.74	TH_HS
1	TH_DB_QL	TH	QL	0	0.50	0.46	0.54	0.00	0.00	-18074.02	-18000.92	1310.00	Ture	73.10	0.80	TH_DB
1	HS_TH_DB	DB	HS	0	0.53	0.40	0.60	0.00	0.00	-19102.82	-18996.69	1381.00	Ture	106.13	0.93	TH_DB
1	HS_TH_DB	HS	HS	0	0.63	0.47	0.53	0.00	0.00	-23292.04	-23173.82	1691.00	False	118.22	1.02	TH_DB
1	TH_DB_QL	QL	QL	0	0.64	0.48	0.52	0.00	0.00	-25395.01	-25261.44	1824.00	False	133.56	1.08	TH_DB
1	HS_DB_QL	DB	QL	0	0.45	0.28	0.72	0.00	0.00	-19473.12	-19322.09	1387.00	Ture	151.04	1.12	HS_DB
1	HS_DB_QL	HS	QL	0	0.45	0.28	0.72	0.00	0.00	-20168.52	-20010.37	1437.00	Ture	158.15	1.17	HS_DB
1	HS_TH_QL	HS	QL	0	0.44	0.27	0.73	0.00	0.00	-19738.70	-19577.14	1416.00	Ture	161.56	1.18	TH_HS
1	HS_DB_QL	QL	QL	0	0.57	0.36	0.64	0.00	0.00	-22812.78	-22643.40	1620.00	False	169.38	1.18	HS_DB

1	HS_TH_QL	QL	QL	0	0.48	0.34	0.66	0.00	0.00	-23034.46	-22853.61	1644.00	False	180.85	1.23	TH_HS
2	HS_DB_TH	DB	HS	0	0.53	0.37	0.63	0.00	0.00	-17083.42	-16969.44	1235.00	Ture	113.98	0.87	DB_TH
2	QL_DB_TH	DB	QL	0	0.49	0.30	0.70	0.00	0.00	-16265.58	-16122.97	1154.00	Ture	142.61	0.91	TH_DB
2	QL_DB_TH	TH	QL	0	0.47	0.30	0.70	0.00	0.00	-16321.60	-16193.03	1181.00	Ture	128.57	0.94	TH_DB
2	HS_QL_TH	TH	QL	0	0.50	0.31	0.69	0.00	0.00	-17432.56	-17299.72	1259.00	Ture	132.84	0.98	TH_HS
2	HS_DB_TH	TH	HS	0	0.47	0.27	0.73	0.00	0.00	-17117.53	-16980.95	1245.00	Ture	136.58	1.02	DB_TH
2	HS_QL_DB	DB	QL	0	0.53	0.26	0.74	0.00	0.00	-17973.35	-17799.35	1268.00	Ture	174.00	1.07	HS_DB
2	QL_DB_TH	QL	QL	0	0.57	0.38	0.62	0.00	0.00	-21711.72	-21564.44	1552.00	False	147.28	1.10	TH_DB
2	HS_QL_TH	HS	QL	0	0.51	0.19	0.81	0.00	0.00	-17036.07	-16848.26	1211.00	Ture	187.81	1.11	TH_HS
2	HS_QL_DB	HS	QL	0	0.47	0.18	0.82	0.00	0.00	-16923.84	-16742.91	1205.00	Ture	180.93	1.11	HS_DB
2	HS_QL_DB	QL	QL	0	0.58	0.30	0.70	0.00	0.00	-20026.25	-19847.08	1414.00	False	179.17	1.12	HS_DB
2	HS_QL_TH	QL	QL	0	0.48	0.25	0.75	0.00	0.00	-19866.27	-19691.18	1417.00	False	175.09	1.20	TH_HS
2	HS_DB_TH	HS	HS	0	0.41	0.21	0.79	0.00	0.00	-19447.17	-19259.63	1407.00	False	187.54	1.26	DB_TH
3	HS_DB_TH	DB	HS	0	0.66	0.45	0.55	0.00	0.00	-15579.67	-15491.26	1128.00	Ture	88.41	0.71	DB_TH
3	HS_DB_TH	HS	HS	0	0.69	0.47	0.53	0.00	0.00	-18213.64	-18113.45	1332.00	False	100.19	0.80	DB_TH
3	HS_QL_TH	TH	QL	0	0.52	0.33	0.67	0.00	0.00	-16428.46	-16311.09	1183.00	Ture	117.37	0.89	TH_HS
3	DB_QL_TH	TH	QL	0	0.59	0.33	0.67	0.00	0.00	-16636.40	-16505.53	1199.00	Ture	130.87	0.90	DB_TH
3	DB_QL_TH	QL	QL	0	0.55	0.40	0.60	0.00	0.00	-19420.80	-19300.89	1399.00	False	119.91	0.95	DB_TH
3	HS_DB_QL	DB	QL	0	0.49	0.23	0.77	0.00	0.00	-15994.07	-15837.30	1123.00	Ture	156.77	0.98	HS_DB
3	HS_DB_TH	TH	HS	0	0.53	0.28	0.72	0.00	0.00	-16686.08	-16540.58	1214.00	Ture	145.50	0.99	DB_TH
3	DB_QL_TH	DB	QL	0	0.44	0.17	0.83	0.00	0.00	-15118.55	-14958.56	1076.00	Ture	159.99	1.02	DB_TH
3	HS_QL_TH	HS	QL	0	0.41	0.22	0.78	0.00	0.00	-16194.20	-16043.18	1169.00	Ture	151.02	1.03	TH_HS
3	HS_DB_QL	HS	QL	0	0.50	0.19	0.81	0.00	0.00	-17012.18	-16824.60	1215.00	Ture	187.58	1.11	HS_DB

3	HS_DB_QL	QL	QL	0	0.59	0.26	0.74	0.00	0.00	-18872.16	-18678.11	1336.00	False	194.05	1.11	HS_DB
3	HS_QL_TH	QL	QL	0	0.44	0.16	0.84	0.00	0.00	-18518.96	-18315.49	1322.00	False	203.47	1.25	TH_HS
4	DB_QL_TH	TH	QL	0	0.55	0.53	0.47	0.00	0.00	-13840.35	-13793.91	1022.00	Ture	46.44	0.54	DB_TH
4	HS_DB_QL	HS	QL	0	0.59	0.49	0.51	0.00	0.00	-14300.29	-14239.78	1047.00	Ture	60.51	0.60	HS_DB
4	HS_QL_TH	TH	QL	0	0.48	0.42	0.58	0.00	0.00	-14795.30	-14730.35	1089.00	Ture	64.95	0.71	TH_HS
4	HS_QL_TH	HS	QL	0	0.49	0.29	0.71	0.00	0.00	-14479.18	-14356.35	1054.00	Ture	122.83	0.85	TH_HS
4	DB_QL_TH	DB	QL	0	0.43	0.24	0.76	0.00	0.00	-14126.02	-14010.03	1012.00	Ture	116.00	0.88	DB_TH
4	HS_DB_TH	TH	HS	0	0.42	0.26	0.74	0.00	0.00	-14594.91	-14483.99	1080.00	Ture	110.92	0.91	DB_TH
4	HS_DB_QL	DB	QL	0	0.42	0.22	0.78	0.00	0.00	-14849.68	-14720.17	1065.00	Ture	129.52	0.94	HS_DB
4	HS_DB_TH	HS	HS	0	0.45	0.30	0.70	0.00	0.00	-16211.61	-16098.46	1200.00	False	113.15	0.95	DB_TH
4	HS_DB_TH	DB	HS	0	0.39	0.22	0.78	0.00	0.00	-14827.15	-14702.02	1088.00	Ture	125.13	0.96	DB_TH
4	DB_QL_TH	QL	QL	0	0.43	0.30	0.70	0.00	0.00	-18180.07	-18060.57	1334.00	False	119.50	1.06	DB_TH
4	HS_QL_TH	QL	QL	0	0.34	0.23	0.77	0.00	0.00	-16551.81	-16434.22	1225.00	False	117.59	1.07	TH_HS
4	HS_DB_QL	QL	QL	0	0.36	0.16	0.84	0.00	0.00	-17318.68	-17160.56	1256.00	False	158.11	1.19	HS_DB
5	HS_DB_TH	HS	HS	0	0.64	0.51	0.49	0.00	0.00	-18235.80	-18151.20	1357.00	False	84.59	0.76	DB_TH
5	DB_QL_TH	DB	QL	0	0.40	0.18	0.82	0.00	0.00	-15090.02	-14940.97	1091.00	Ture	149.06	1.02	DB_TH
5	DB_QL_TH	TH	QL	0	0.49	0.17	0.83	0.00	0.00	-15881.25	-15689.31	1146.00	Ture	191.94	1.07	DB_TH
5	HS_QL_TH	TH	QL	0	0.46	0.19	0.81	0.00	0.00	-16593.88	-16409.68	1204.00	Ture	184.20	1.11	TH_HS
5	HS_QL_TH	HS	QL	0	0.48	0.17	0.83	0.00	0.00	-16823.37	-16629.15	1217.00	Ture	194.22	1.14	TH_HS
5	HS_DB_TH	TH	HS	0	0.44	0.15	0.85	0.00	0.00	-16605.44	-16398.89	1208.00	Ture	206.55	1.16	DB_TH
5	HS_DB_TH	DB	HS	0	0.42	0.11	0.89	0.00	0.00	-15927.17	-15724.68	1154.00	Ture	202.49	1.16	DB_TH
5	HS_DB_QL	DB	QL	0	0.45	0.10	0.90	0.00	0.00	-16527.03	-16302.85	1183.00	Ture	224.18	1.21	HS_DB
5	HS_DB_QL	HS	QL	0	0.43	0.09	0.91	0.00	0.00	-16803.19	-16579.23	1207.00	Ture	223.96	1.24	HS_DB
5	HS_DB_QL	QL	QL	0	0.47	0.15	0.85	0.00	0.00	-18598.87	-18375.39	1329.00	False	223.47	1.27	HS_DB
5	HS_QL_TH	QL	QL	0	0.40	0.12	0.88	0.00	0.00	-18063.25	-17838.45	1298.00	False	224.80	1.29	TH_HS
5	DB_QL_TH	QL	QL	0	0.39	0.11	0.89	0.00	0.00	-20480.54	-20222.04	1482.00	False	258.51	1.50	DB_TH

6	HS_QL_TH	TH	QL	0	0.96	0.76	0.24	0.00	0.00	-15645.17	-15638.42	1141.00	Ture	6.76	0.31	TH_HS
6	DB_QL_TH	TH	QL	0	0.63	0.46	0.54	0.00	0.00	-14923.75	-14848.10	1076.00	Ture	75.65	0.66	DB_TH
6	DB_QL_TH	QL	QL	0	0.66	0.55	0.45	0.00	0.00	-19826.89	-19759.04	1429.00	False	67.85	0.73	DB_TH
6	HS_DB_QL	DB	QL	0	0.53	0.33	0.67	0.00	0.00	-15977.24	-15864.43	1131.00	Ture	112.81	0.86	HS_DB
6	HS_QL_TH	HS	QL	0	0.56	0.27	0.73	0.00	0.00	-15555.17	-15407.61	1117.00	Ture	147.56	0.93	TH_HS
6	HS_DB_TH	TH	HS	0	0.44	0.25	0.75	0.00	0.00	-15136.34	-15009.87	1108.00	Ture	126.47	0.93	DB_TH
6	HS_DB_TH	DB	HS	0	0.51	0.26	0.74	0.00	0.00	-15834.50	-15691.27	1133.00	Ture	143.23	0.95	DB_TH
6	DB_QL_TH	DB	QL	0	0.52	0.20	0.80	0.00	0.00	-14845.50	-14681.06	1050.00	Ture	164.44	0.95	DB_TH
6	HS_DB_QL	HS	QL	0	0.56	0.23	0.77	0.00	0.00	-15675.62	-15500.98	1114.00	Ture	174.64	0.96	HS_DB
6	HS_QL_TH	QL	QL	0	0.46	0.29	0.71	0.00	0.00	-18223.06	-18059.60	1297.00	False	163.46	1.05	TH_HS
6	HS_DB_QL	QL	QL	0	0.50	0.27	0.73	0.00	0.00	-18511.42	-18341.47	1310.00	False	169.95	1.08	HS_DB
6	HS_DB_TH	HS	HS	0	0.45	0.23	0.77	0.00	0.00	-18023.17	-17859.96	1314.00	False	163.21	1.14	DB_TH
7	QL_DB_TH	DB	QL	0	0.79	0.67	0.33	0.00	0.00	-13648.31	-13629.12	983.00	Ture	19.19	0.37	TH_DB
7	HS_QL_TH	HS	QL	0	0.75	0.65	0.35	0.00	0.00	-14215.52	-14188.15	1027.00	Ture	27.37	0.41	TH_HS
7	HS_QL_TH	QL	QL	0	0.73	0.68	0.32	0.00	0.00	-16906.45	-16889.45	1237.00	False	17.00	0.45	TH_HS
7	HS_DB_TH	TH	HS	0	0.60	0.62	0.38	0.00	0.00	-15008.75	-14983.89	1109.00	Ture	24.85	0.47	DB_TH
7	HS_QL_TH	TH	QL	0	0.52	0.46	0.54	0.00	0.00	-15265.96	-15205.88	1106.00	Ture	60.08	0.68	TH_HS
7	QL_DB_TH	QL	QL	0	0.56	0.50	0.50	0.00	0.00	-17957.13	-17891.12	1310.00	False	66.00	0.74	TH_DB
7	HS_QL_DB	DB	QL	0	0.41	0.35	0.65	0.00	0.00	-14725.85	-14619.89	1047.00	Ture	105.96	0.77	HS_DB
7	QL_DB_TH	TH	QL	0	0.41	0.30	0.70	0.00	0.00	-14867.01	-14758.91	1077.00	Ture	108.10	0.86	TH_DB
7	HS_DB_TH	HS	HS	0	0.48	0.32	0.68	0.00	0.00	-15810.41	-15707.28	1159.00	False	103.13	0.89	DB_TH
7	HS_QL_DB	HS	QL	0	0.43	0.22	0.78	0.00	0.00	-14940.48	-14804.82	1067.00	Ture	135.65	0.94	HS_DB
7	HS_QL_DB	QL	QL	0	0.42	0.31	0.69	0.00	0.00	-17409.09	-17297.13	1256.00	False	111.95	0.98	HS_DB
7	HS_DB_TH	DB	HS	0	0.36	0.21	0.79	0.00	0.00	-15208.85	-15079.23	1102.00	Ture	129.62	0.98	DB_TH
8	DB_TH_QL	TH	QL	0	0.75	0.61	0.39	0.00	0.00	-11959.01	-11933.13	868.00	Ture	25.88	0.38	DB_TH
8	HS_TH_QL	TH	QL	0	0.56	0.40	0.60	0.00	0.00	-12845.57	-12770.38	935.00	Ture	75.20	0.63	TH_HS

8	HS_DB_TH	TH	HS	0	0.51	0.31	0.69	0.00	0.00	-12299.80	-12207.26	895.00	Ture	92.55	0.69	DB_TH
8	HS_DB_TH	DB	HS	0	0.57	0.34	0.66	0.00	0.00	-13071.21	-12968.35	949.00	Ture	102.86	0.71	DB_TH
8	HS_DB_QL	HS	QL	0	0.53	0.25	0.75	0.00	0.00	-12544.44	-12422.03	897.00	Ture	122.41	0.76	HS_DB
8	DB_TH_QL	DB	QL	0	0.45	0.21	0.79	0.00	0.00	-12453.33	-12336.76	888.00	Ture	116.57	0.80	DB_TH
8	HS_DB_TH	HS	HS	0	0.48	0.28	0.72	0.00	0.00	-13878.63	-13765.03	1019.00	False	113.60	0.83	DB_TH
8	HS_DB_QL	QL	QL	0	0.53	0.25	0.75	0.00	0.00	-14212.87	-14077.89	1008.00	False	134.99	0.86	HS_DB
8	HS_TH_QL	HS	QL	0	0.41	0.12	0.88	0.00	0.00	-12028.90	-11883.13	867.00	Ture	145.76	0.87	TH_HS
8	HS_DB_QL	DB	QL	0	0.45	0.15	0.85	0.00	0.00	-13438.29	-13282.40	958.00	Ture	155.88	0.92	HS_DB
8	DB_TH_QL	QL	QL	0	0.43	0.23	0.77	0.00	0.00	-15320.04	-15188.10	1107.00	False	131.93	0.96	DB_TH
8	HS_TH_QL	QL	QL	0	0.44	0.12	0.88	0.00	0.00	-14844.58	-14665.76	1061.00	False	178.82	1.05	TH_HS
9	HS_QL_TH	QL	QL	0	0.72	0.55	0.45	0.00	0.00	-12637.99	-12591.47	907.00	False	46.52	0.46	TH_HS
9	DB_QL_TH	QL	QL	0	0.77	0.57	0.43	0.00	0.00	-14340.41	-14287.29	1033.00	False	53.12	0.50	DB_TH
9	HS_DB_QL	QL	QL	0	0.68	0.45	0.55	0.00	0.00	-12879.05	-12806.28	917.00	False	72.76	0.58	HS_DB
9	HS_DB_TH	TH	HS	0	0.53	0.37	0.63	0.00	0.00	-11597.48	-11529.30	844.00	Ture	68.18	0.60	DB_TH
9	DB_QL_TH	TH	QL	0	0.48	0.35	0.65	0.00	0.00	-11479.95	-11410.98	829.00	Ture	68.97	0.61	DB_TH
9	HS_QL_TH	TH	QL	0	0.48	0.40	0.60	0.00	0.00	-12275.23	-12209.82	890.00	Ture	65.41	0.61	TH_HS
9	DB_QL_TH	DB	QL	0	0.45	0.25	0.75	0.00	0.00	-10838.98	-10752.94	780.00	Ture	86.04	0.66	DB_TH
9	HS_DB_QL	DB	QL	0	0.49	0.33	0.67	0.00	0.00	-12413.91	-12315.27	885.00	Ture	98.64	0.67	HS_DB
9	HS_DB_QL	HS	QL	0	0.44	0.24	0.76	0.00	0.00	-11700.25	-11597.52	840.00	Ture	102.72	0.72	HS_DB
9	HS_DB_TH	DB	HS	0	0.44	0.21	0.79	0.00	0.00	-11352.02	-11245.59	818.00	Ture	106.43	0.73	DB_TH
9	HS_DB_TH	HS	HS	0	0.53	0.34	0.66	0.00	0.00	-13442.57	-13349.11	980.00	False	93.45	0.73	DB_TH
9	HS_QL_TH	HS	QL	0	0.42	0.17	0.83	0.00	0.00	-11731.62	-11610.92	845.00	Ture	120.70	0.79	TH_HS
10	HS_TH_QL	TH	QL	0	0.47	0.33	0.67	0.00	0.00	-8738.27	-8687.30	637.00	Ture	50.97	0.49	TH_HS
10	DB_TH_QL	TH	QL	0	0.45	0.26	0.74	0.00	0.00	-8072.74	-8011.22	589.00	Ture	61.52	0.49	DB_TH
10	HS_DB_TH	TH	HS	0	0.45	0.26	0.74	0.00	0.00	-8296.72	-8225.87	608.00	Ture	70.85	0.51	DB_TH
10	DB_TH_QL	DB	QL	0	0.43	0.24	0.76	0.00	0.00	-8220.58	-8157.50	591.00	Ture	63.08	0.51	DB_TH

10	HS_DB_QL	DB	QL	0	0.41	0.25	0.75	0.00	0.00	-8760.70	-8698.93	628.00	Ture	61.77	0.53	HS_DB
10	HS_TH_QL	HS	QL	0	0.49	0.18	0.82	0.00	0.00	-8438.27	-8350.81	603.00	Ture	87.46	0.56	TH_HS
10	HS_DB_QL	HS	QL	0	0.51	0.14	0.86	0.00	0.00	-8673.24	-8564.86	614.00	Ture	108.39	0.59	HS_DB
10	HS_DB_TH	DB	HS	0	0.36	0.13	0.87	0.00	0.00	-8593.38	-8503.20	626.00	Ture	90.18	0.62	DB_TH
10	HS_DB_QL	QL	QL	0	0.49	0.18	0.82	0.00	0.00	-9498.61	-9396.17	675.00	False	102.44	0.63	HS_DB
10	HS_TH_QL	QL	QL	0	0.38	0.13	0.87	0.00	0.00	-9367.40	-9266.60	677.00	False	100.80	0.67	TH_HS
10	HS_DB_TH	HS	HS	0	0.35	0.03	0.97	0.00	0.00	-9500.85	-9360.70	683.00	False	140.15	0.75	DB_TH
10	DB_TH_QL	QL	QL	0	0.40	0.08	0.92	0.00	0.00	-10311.13	-10172.13	737.00	False	139.00	0.77	DB_TH
11	DB_QL_TH	DB	QL	0	0.77	0.57	0.43	0.00	0.00	-11039.21	-11002.59	785.00	Ture	36.62	0.38	DB_TH
11	HS_DB_TH	TH	HS	0	0.67	0.56	0.44	0.00	0.00	-11919.48	-11884.40	883.00	Ture	35.09	0.44	DB_TH
11	DB_QL_TH	QL	QL	0	0.85	0.58	0.42	0.00	0.00	-13911.38	-13855.58	1003.00	False	55.80	0.47	DB_TH
11	HS_DB_QL	DB	QL	0	0.74	0.44	0.56	0.00	0.00	-11399.30	-11325.77	808.00	Ture	73.54	0.52	HS_DB
11	HS_QL_TH	HS	QL	0	0.57	0.35	0.65	0.00	0.00	-12089.33	-12005.21	861.00	Ture	84.11	0.63	TH_HS
11	HS_DB_TH	DB	HS	0	0.51	0.27	0.73	0.00	0.00	-10970.02	-10877.70	794.00	Ture	92.32	0.66	DB_TH
11	HS_DB_QL	HS	QL	0	0.59	0.29	0.71	0.00	0.00	-11876.31	-11767.82	845.00	Ture	108.49	0.68	HS_DB
11	HS_DB_QL	QL	QL	0	0.58	0.34	0.66	0.00	0.00	-13848.47	-13734.79	971.00	False	113.68	0.72	HS_DB
11	HS_QL_TH	TH	QL	0	0.39	0.24	0.76	0.00	0.00	-11504.95	-11415.94	844.00	Ture	89.01	0.73	TH_HS
11	DB_QL_TH	TH	QL	0	0.44	0.22	0.78	0.00	0.00	-11448.35	-11341.93	836.00	Ture	106.41	0.74	DB_TH
11	HS_QL_TH	QL	QL	0	0.46	0.15	0.85	0.00	0.00	-12976.91	-12830.53	919.00	False	146.38	0.88	TH_HS
11	HS_DB_TH	HS	HS	0	0.46	0.15	0.85	0.00	0.00	-12971.37	-12820.16	947.00	False	151.21	0.91	DB_TH
12	DB_QL_TH	DB	QL	0	2.19	0.96	0.04	0.00	0.00	-11247.43	-11270.00	800.00	Ture	-22.57	0.03	DB_TH
12	HS_DB_QL	HS	QL	0	0.86	0.66	0.34	0.00	0.00	-11742.85	-11721.21	836.00	Ture	21.63	0.32	HS_DB
12	HS_DB_QL	DB	QL	0	0.72	0.50	0.50	0.00	0.00	-11629.67	-11577.30	826.00	Ture	52.37	0.47	HS_DB
12	DB_QL_TH	TH	QL	0	0.55	0.40	0.60	0.00	0.00	-10680.62	-10622.31	770.00	Ture	58.31	0.52	DB_TH
12	HS_QL_TH	TH	QL	0	0.57	0.38	0.62	0.00	0.00	-11439.15	-11370.44	829.00	Ture	68.71	0.58	TH_HS
12	HS_DB_TH	HS	HS	0	0.63	0.45	0.55	0.00	0.00	-13075.17	-13003.39	952.00	False	71.78	0.59	DB_TH

12	HS_DB_TH	DB	HS	0	0.51	0.32	0.68	0.00	0.00	-11051.87	-10968.69	788.00	Ture	83.17	0.60	DB_TH
12	HS_QL_TH	QL	QL	0	0.60	0.38	0.62	0.00	0.00	-12603.55	-12524.31	892.00	False	79.23	0.62	TH_HS
12	HS_QL_TH	HS	QL	0	0.50	0.34	0.66	0.00	0.00	-12023.63	-11943.64	856.00	Ture	79.99	0.64	TH_HS
12	HS_DB_TH	TH	HS	0	0.43	0.26	0.74	0.00	0.00	-11635.49	-11530.79	837.00	Ture	104.70	0.70	DB_TH
12	DB_QL_TH	QL	QL	0	0.51	0.37	0.63	0.00	0.00	-14065.43	-13971.95	1007.00	False	93.48	0.71	DB_TH
12	HS_DB_QL	QL	QL	0	0.49	0.22	0.78	0.00	0.00	-13061.32	-12936.93	915.00	False	124.40	0.81	HS_DB
13	HS_DB_TH	TH	HS	0	0.72	0.63	0.37	0.00	0.00	-9208.69	-9192.76	677.00	Ture	15.93	0.28	DB_TH
13	HS_QL_DB	DB	QL	0	0.73	0.62	0.38	0.00	0.00	-9974.93	-9955.11	709.00	Ture	19.83	0.30	HS_DB
13	HS_QL_TH	TH	QL	0	0.71	0.59	0.41	0.00	0.00	-9695.43	-9671.99	705.00	Ture	23.44	0.32	TH_HS
13	QL_DB_TH	TH	QL	0	0.72	0.56	0.44	0.00	0.00	-9391.09	-9363.37	678.00	Ture	27.72	0.34	TH_DB
13	HS_QL_DB	QL	QL	0	0.64	0.50	0.50	0.00	0.00	-10364.29	-10326.34	743.00	False	37.95	0.42	HS_DB
13	QL_DB_TH	DB	QL	0	0.59	0.39	0.61	0.00	0.00	-8814.07	-8759.38	627.00	Ture	54.69	0.43	TH_DB
13	QL_DB_TH	QL	QL	0	0.67	0.50	0.50	0.00	0.00	-11738.51	-11688.73	847.00	False	49.78	0.48	TH_DB
13	HS_DB_TH	HS	HS	0	0.60	0.43	0.57	0.00	0.00	-10532.64	-10477.47	774.00	False	55.18	0.50	DB_TH
13	HS_QL_DB	HS	QL	0	0.51	0.29	0.71	0.00	0.00	-9813.32	-9739.04	700.00	Ture	74.28	0.56	HS_DB
13	HS_QL_TH	HS	QL	0	0.48	0.24	0.76	0.00	0.00	-9294.48	-9217.58	668.00	Ture	76.90	0.58	TH_HS
13	HS_DB_TH	DB	HS	0	0.51	0.27	0.73	0.00	0.00	-9810.87	-9727.75	701.00	Ture	83.12	0.58	DB_TH
13	HS_QL_TH	QL	QL	0	0.50	0.23	0.77	0.00	0.00	-10953.21	-10848.68	779.00	False	104.53	0.68	TH_HS
14	HS_DB_TH	TH	HS	0	5.53	0.99	0.01	0.00	0.00	-10913.88	-10925.15	802.00	Ture	-11.26	0.01	DB_TH
14	HS_TH_QL	QL	QL	0	0.85	0.69	0.31	0.00	0.00	-13543.48	-13522.28	976.00	False	21.19	0.34	TH_HS
14	HS_TH_QL	TH	QL	0	0.70	0.61	0.39	0.00	0.00	-10930.26	-10907.05	792.00	Ture	23.21	0.35	TH_HS
14	HS_DB_TH	HS	HS	0	0.78	0.65	0.35	0.00	0.00	-12699.91	-12678.09	933.00	False	21.82	0.37	DB_TH
14	HS_DB_QL	DB	QL	0	0.73	0.59	0.41	0.00	0.00	-11126.06	-11098.00	791.00	Ture	28.06	0.37	HS_DB
14	HS_TH_QL	HS	QL	0	0.70	0.56	0.44	0.00	0.00	-10572.13	-10538.94	765.00	Ture	33.19	0.38	TH_HS
14	DB_TH_QL	TH	QL	0	0.52	0.44	0.56	0.00	0.00	-10085.18	-10041.06	728.00	Ture	44.13	0.46	DB_TH
14	HS_DB_TH	DB	HS	0	0.48	0.42	0.58	0.00	0.00	-11030.67	-10981.25	798.00	Ture	49.42	0.52	DB_TH

14	DB_TH_QL	DB	QL	0	0.55	0.38	0.62	0.00	0.00	-10542.58	-10477.75	745.00	Ture	64.83	0.53	DB_TH
14	HS_DB_QL	HS	QL	0	0.54	0.34	0.66	0.00	0.00	-11038.10	-10961.18	789.00	Ture	76.92	0.59	HS_DB
14	DB_TH_QL	QL	QL	0	0.61	0.42	0.58	0.00	0.00	-14739.97	-14644.29	1060.00	False	95.68	0.69	DB_TH
14	HS_DB_QL	QL	QL	0	0.47	0.34	0.66	0.00	0.00	-13310.97	-13230.88	953.00	False	80.09	0.72	HS_DB
15	DB_TH_QL	TH	QL	0	7.14	1.00	0.00	0.00	0.00	-9028.95	-9041.92	659.00	Ture	-12.97	0.00	DB_TH
15	HS_TH_QL	HS	QL	0	1.25	0.80	0.20	0.00	0.00	-9489.06	-9488.64	679.00	Ture	0.42	0.15	TH_HS
15	HS_DB_TH	TH	HS	0	0.71	0.65	0.35	0.00	0.00	-9302.68	-9291.07	680.00	Ture	11.60	0.27	DB_TH
15	HS_DB_TH	HS	HS	0	0.89	0.70	0.30	0.00	0.00	-11245.06	-11228.87	820.00	False	16.19	0.28	DB_TH
15	DB_TH_QL	DB	QL	0	0.81	0.58	0.42	0.00	0.00	-8830.76	-8802.14	626.00	Ture	28.61	0.30	DB_TH
15	HS_TH_QL	TH	QL	0	0.74	0.61	0.39	0.00	0.00	-9908.53	-9885.74	714.00	Ture	22.79	0.31	TH_HS
15	HS_DB_QL	HS	QL	0	0.78	0.55	0.45	0.00	0.00	-10292.71	-10252.76	731.00	Ture	39.95	0.37	HS_DB
15	HS_DB_QL	DB	QL	0	0.60	0.48	0.52	0.00	0.00	-9747.48	-9699.81	688.00	Ture	47.67	0.41	HS_DB
15	HS_TH_QL	QL	QL	0	0.63	0.52	0.48	0.00	0.00	-11381.14	-11342.28	816.00	False	38.85	0.44	TH_HS
15	HS_DB_TH	DB	HS	0	0.60	0.44	0.56	0.00	0.00	-9850.09	-9799.54	709.00	Ture	50.55	0.45	DB_TH
15	HS_DB_QL	QL	QL	0	0.61	0.43	0.57	0.00	0.00	-11157.83	-11098.61	790.00	False	59.21	0.51	HS_DB
15	DB_TH_QL	QL	QL	0	0.47	0.28	0.72	0.00	0.00	-12967.92	-12869.60	924.00	False	98.32	0.75	DB_TH
16	HS_QL_TH	TH	QL	0	0.76	0.67	0.33	0.00	0.00	-6722.48	-6717.22	495.00	Ture	5.26	0.19	TH_HS
16	DB_QL_TH	TH	QL	0	0.69	0.61	0.39	0.00	0.00	-6496.06	-6487.35	472.00	Ture	8.72	0.21	DB_TH
16	HS_DB_QL	HS	QL	0	0.74	0.49	0.51	0.00	0.00	-6255.09	-6228.70	442.00	Ture	26.39	0.26	HS_DB
16	HS_DB_TH	DB	HS	0	0.80	0.55	0.45	0.00	0.00	-7077.33	-7052.73	507.00	Ture	24.61	0.26	DB_TH
16	HS_DB_TH	TH	HS	0	0.56	0.55	0.45	0.00	0.00	-6966.82	-6955.45	517.00	Ture	11.37	0.26	DB_TH
16	HS_QL_TH	HS	QL	0	0.42	0.24	0.76	0.00	0.00	-6483.49	-6436.62	463.00	Ture	46.87	0.40	TH_HS
16	DB_QL_TH	DB	QL	0	0.44	0.19	0.81	0.00	0.00	-6702.82	-6644.04	476.00	Ture	58.77	0.44	DB_TH
16	HS_DB_TH	HS	HS	0	0.55	0.20	0.80	0.00	0.00	-7299.63	-7220.00	524.00	False	79.63	0.47	DB_TH
16	HS_DB_QL	DB	QL	0	0.46	0.15	0.85	0.00	0.00	-7253.15	-7178.00	511.00	Ture	75.15	0.49	HS_DB
16	HS_DB_QL	QL	QL	0	0.46	0.19	0.81	0.00	0.00	-8323.72	-8245.06	595.00	False	78.66	0.54	HS_DB

16	DB_QL_TH	QL	QL	0	0.45	0.16	0.84	0.00	0.00	-8381.64	-8287.03	600.00	False	94.61	0.57	DB_TH
16	HS_QL_TH	QL	QL	0	0.43	0.10	0.90	0.00	0.00	-8334.85	-8232.05	590.00	False	102.81	0.60	TH_HS
17	DB_TH_QL	DB	QL	0	0.43	0.10	0.90	0.00	0.00	-7501.63	-7406.14	539.00	Ture	95.48	0.55	DB_TH
17	HS_DB_QL	DB	QL	0	0.49	0.14	0.86	0.00	0.00	-7937.08	-7840.85	571.00	Ture	96.23	0.56	HS_DB
17	HS_DB_TH	TH	HS	0	0.43	0.13	0.87	0.00	0.00	-7894.32	-7800.52	584.00	Ture	93.80	0.58	DB_TH
17	DB_TH_QL	TH	QL	0	0.39	0.09	0.91	0.00	0.00	-7691.62	-7598.71	561.00	Ture	92.90	0.58	DB_TH
17	HS_DB_TH	DB	HS	0	0.43	0.10	0.90	0.00	0.00	-8292.68	-8190.16	605.00	Ture	102.52	0.61	DB_TH
17	HS_TH_QL	TH	QL	0	0.40	0.05	0.95	0.00	0.00	-7885.28	-7767.26	575.00	Ture	118.02	0.62	TH_HS
17	HS_DB_QL	QL	QL	0	0.49	0.18	0.82	0.00	0.00	-9350.63	-9249.47	676.00	False	101.17	0.63	HS_DB
17	HS_TH_QL	QL	QL	0	0.46	0.19	0.81	0.00	0.00	-9579.72	-9480.62	695.00	False	99.10	0.64	TH_HS
17	HS_TH_QL	HS	QL	0	0.46	0.05	0.95	0.00	0.00	-8426.04	-8289.03	610.00	Ture	137.01	0.66	TH_HS
17	HS_DB_QL	HS	QL	0	0.44	0.05	0.95	0.00	0.00	-8774.04	-8634.46	633.00	Ture	139.58	0.68	HS_DB
17	HS_DB_TH	HS	HS	0	0.38	0.06	0.94	0.00	0.00	-9285.58	-9148.72	691.00	False	136.86	0.74	DB_TH
17	DB_TH_QL	QL	QL	0	0.40	0.08	0.92	0.00	0.00	-10773.99	-10622.72	780.00	False	151.28	0.81	DB_TH
18	QL_DB_TH	TH	QL	0	0.46	0.04	0.96	0.00	0.00	-5367.18	-5282.74	386.00	Ture	84.44	0.42	TH_DB
18	HS_QL_TH	QL	QL	0	0.47	0.26	0.74	0.00	0.00	-7151.52	-7095.64	522.00	False	55.88	0.44	TH_HS
18	HS_DB_TH	TH	HS	0	0.41	0.04	0.96	0.00	0.00	-5868.14	-5781.42	426.00	Ture	86.72	0.46	DB_TH
18	HS_QL_DB	HS	QL	0	0.47	0.07	0.93	0.00	0.00	-6270.57	-6177.64	449.00	Ture	92.93	0.47	HS_DB
18	HS_QL_TH	TH	QL	0	0.46	0.04	0.96	0.00	0.00	-6268.81	-6165.59	450.00	Ture	103.22	0.49	TH_HS
18	HS_QL_DB	DB	QL	0	0.41	0.05	0.95	0.00	0.00	-6461.32	-6370.77	463.00	Ture	90.55	0.50	HS_DB
18	HS_QL_TH	HS	QL	0	0.39	0.09	0.91	0.00	0.00	-6672.18	-6586.05	484.00	Ture	86.13	0.50	TH_HS
18	HS_DB_TH	DB	HS	0	0.41	0.04	0.96	0.00	0.00	-6484.62	-6389.10	472.00	Ture	95.52	0.51	DB_TH
18	QL_DB_TH	DB	QL	0	0.42	0.02	0.98	0.00	0.00	-6570.96	-6461.07	470.00	Ture	109.89	0.52	TH_DB
18	HS_QL_DB	QL	QL	0	0.51	0.12	0.88	0.00	0.00	-7667.11	-7561.99	544.00	False	105.12	0.54	HS_DB
18	HS_DB_TH	HS	HS	0	0.41	0.05	0.95	0.00	0.00	-7656.06	-7541.97	558.00	False	114.09	0.60	DB_TH
18	QL_DB_TH	QL	QL	0	0.42	0.06	0.94	0.00	0.00	-8334.90	-8215.36	600.00	False	119.54	0.64	TH_DB

19	HS_DB_QL	HS	QL	0	0.56	0.32	0.68	0.00	0.00	-5106.26	-5071.94	374.00	Ture	34.32	0.29	HS_DB
19	HS_DB_QL	DB	QL	0	0.64	0.31	0.69	0.00	0.00	-5053.05	-5014.27	370.00	Ture	38.78	0.29	HS_DB
19	HS_QL_TH	QL	QL	0	0.45	0.22	0.78	0.00	0.00	-5017.67	-4968.05	370.00	False	49.62	0.33	TH_HS
19	DB_QL_TH	DB	QL	0	0.51	0.16	0.84	0.00	0.00	-4841.84	-4791.34	352.00	Ture	50.50	0.33	DB_TH
19	HS_DB_TH	TH	HS	0	0.50	0.17	0.83	0.00	0.00	-4906.42	-4856.98	370.00	Ture	49.44	0.35	DB_TH
19	HS_DB_TH	DB	HS	0	0.43	0.09	0.91	0.00	0.00	-4649.97	-4591.81	343.00	Ture	58.16	0.35	DB_TH
19	DB_QL_TH	QL	QL	0	0.51	0.20	0.80	0.00	0.00	-5409.74	-5356.76	405.00	False	52.99	0.37	DB_TH
19	DB_QL_TH	TH	QL	0	0.41	0.10	0.90	0.00	0.00	-4893.16	-4836.42	366.00	Ture	56.74	0.37	DB_TH
19	HS_QL_TH	HS	QL	0	0.40	0.08	0.92	0.00	0.00	-4895.12	-4835.13	360.00	Ture	59.99	0.37	TH_HS
19	HS_DB_TH	HS	HS	0	0.48	0.19	0.81	0.00	0.00	-5451.90	-5400.46	410.00	False	51.43	0.38	DB_TH
19	HS_DB_QL	QL	QL	0	0.49	0.09	0.91	0.00	0.00	-5214.19	-5139.77	379.00	False	74.42	0.39	HS_DB
19	HS_QL_TH	TH	QL	0	0.35	0.04	0.96	0.00	0.00	-5249.03	-5182.48	393.00	Ture	66.55	0.42	TH_HS
20	HS_TH_QL	TH	QL	0	0.79	0.56	0.44	0.00	0.00	-6635.64	-6614.05	483.00	Ture	21.59	0.24	TH_HS
20	TH_DB_QL	TH	QL	0	0.60	0.37	0.63	0.00	0.00	-6187.19	-6148.30	449.00	Ture	38.88	0.32	TH_DB
20	HS_TH_DB	DB	HS	0	0.52	0.17	0.83	0.00	0.00	-6078.00	-6008.47	441.00	Ture	69.53	0.42	TH_DB
20	TH_DB_QL	DB	QL	0	0.48	0.11	0.89	0.00	0.00	-6020.00	-5940.24	427.00	Ture	79.76	0.43	TH_DB
20	HS_DB_QL	DB	QL	0	0.55	0.18	0.82	0.00	0.00	-6642.71	-6565.84	466.00	Ture	76.87	0.43	HS_DB
20	HS_DB_QL	HS	QL	0	0.59	0.12	0.88	0.00	0.00	-6453.56	-6357.92	462.00	Ture	95.64	0.46	HS_DB
20	HS_TH_DB	TH	HS	0	0.39	0.18	0.82	0.00	0.00	-6816.50	-6753.64	498.00	Ture	62.86	0.46	TH_DB
20	HS_TH_QL	HS	QL	0	0.53	0.17	0.83	0.00	0.00	-6853.16	-6775.39	493.00	Ture	77.77	0.46	TH_HS
20	HS_TH_QL	QL	QL	0	0.46	0.13	0.87	0.00	0.00	-7392.79	-7305.11	534.00	False	87.69	0.53	TH_HS
20	HS_DB_QL	QL	QL	0	0.50	0.14	0.86	0.00	0.00	-8217.51	-8117.70	582.00	False	99.81	0.56	HS_DB
20	TH_DB_QL	QL	QL	0	0.53	0.20	0.80	0.00	0.00	-8808.83	-8705.70	634.00	False	103.13	0.58	TH_DB
20	HS_TH_DB	HS	HS	0	0.42	0.07	0.93	0.00	0.00	-7802.67	-7690.45	571.00	False	112.21	0.60	TH_DB

Table S4. The results of likelihood-ration tests and AIC comparisons for nine demographic models.

Models	P	logL	AIC
Model 1	13	-1402.16	2830.32
Model 2	15	-1402.15	2834.3
Model 3	17	-1376.47	2786.52
Model 4	19	-1376.02	2789.52
Model 5	21	-1316.9	2674.14
Model 6	23	-1317.19	2678.7
Model 7	25	-1316.57	2681.92
Model 8	27	-1339.24	2726.78
Model 9	29	-1337.95	2728.98

Table S5. Demographic parameters inferred by Generalized Phylogenetic Coalescent Sampler (G-PhoCS) software.

Parapeters	Mean	Upper 95% CI	lower 95% CI
θ_{TH}	1182.68	1183.06	1182.31
θ_{DB}	1382.39	1382.83	1381.94
θ_{HS}	3534.52	3535.52	3533.53
θ_{QL}	10902.06	10903.19	10900.93
θ_{THDB}	40905.44	40912.99	40897.88
θ_{HSDBTH}	186.38	186.50	186.25
θ_{QL_HSDBTH}	159.57	159.66	159.47
T_{THDB}	11583.69	11587.34	11580.04
T_{HSDBTH}	128498.94	128507.45	128490.42
T_{QL_HSDBTH}	300783.51	300802.73	300764.29
$M_{TH->DB}$	0.33	0.30	0.32
$M_{DB->TH}$	0.33	0.30	0.32
$M_{HS->THDB}$	0.04	0.04	0.04
$M_{THDB->HS}$	1655.64	1656.53	1654.76
$M_{HSDBTH->QL}$	0.03	0.03	0.03
$M_{QL->HSDBTH}$	756355.70	757714.90	754996.60
$M_{HS->DB}$	0.27	0.27	0.26
$M_{DB->HS}$	0.27	0.28	0.27

Table S6. G-PhoCS results from three independent results.

Parapeters	Rep1	Rep2	Rep3
	Mean (95% CI Lower-95% CI Upper)	Mean (95% CI Lower-95% CI Upper)	Mean (95% CI Lower-95% CI Upper)
θ_{TH}	1184.60 (1184.23 -1184.97)	1174.68 (1174.32-1175.05)	1239.71 (1239.3-1240.13)
θ_{DB}	1418.08 (1417.62 -1418.53)	1440.68 (1440.23-1441.14)	1464.88 (1464.38-1465.38)
θ_{HS}	3731.06 (3729.80 -3732.32)	3552.78 (3551.77-3553.79)	3809.22 (3808.17-3810.26)
θ_{QL}	11361.44 (11360.28 -11362.59)	11048.54 (11047.44-11049.63)	12565.18 (12563.87-12566.49)
θ_{THDB}	41530.13 (41521.76 -41538.49)	40519.61 (40512.43-40526.79)	42457.36 (42450.39-42464.29)
θ_{HSDBTH}	181.05 (180.92 -181.18)	185.73 (185.61-185.85)	179.38 (179.27-179.49)
θ_{QL_HSDBTH}	159.97 (159.87 -160.07)	160.01 (159.91-160.11)	159.59 (159.49-159.69)
T_{THDB}	11744.69 (11740.98 -11748.39)	12453.96 (12450.02-12457.89)	12138.86 (12134.76-12142.95)
T_{HSDBTH}	135747.14 (135738.18 -135756.10)	126336.83 (126328.35-126345.31)	142618.05 (142608.31-142627.79)
T_{QL_HSDBTH}	319794.81 (319774.81 -319814.81)	305135.45 (305116.36-305154.42)	346413.64 (346391.95-346435.19)
$M_{TH->DB}$	0.32 (0.32 -0.33)	0.28 (0.28-0.29)	0.34 (0.33-0.35)
$M_{DB->TH}$	0.33 (0.32 -0.33)	0.3 (0.29-0.3)	0.35 (0.34-0.36)
$M_{HS->THDB}$	0.033 (0.032-0.034)	0.035 (0.03-0.04)	0.038 (0.037-0.038)
$M_{THDB->HS}$	1511.45 (1510.41-1512.51)	1693.50 (1691.60-1693.50)	1343.01 (1341.59-1343.01)
$M_{HSDBTH->QL}$	0.024 (0.024-0.025)	0.024 (0.023-0.024)	0.027 (0.026-0.027)
$M_{QL->HSDBTH}$	1043682 (1040962-1046402)	817654.40 (815192.20-817654.4)	1252576.00 (1248620-1252576)
$M_{HS->DB}$	0.26 (0.26 -0.27)	0.25 (0.25-0.26)	0.28 (0.27-0.28)
$M_{DB->HS}$	0.27 (0.27-0.28)	0.24 (0.24-0.25)	0.28 (0.27-0.28)

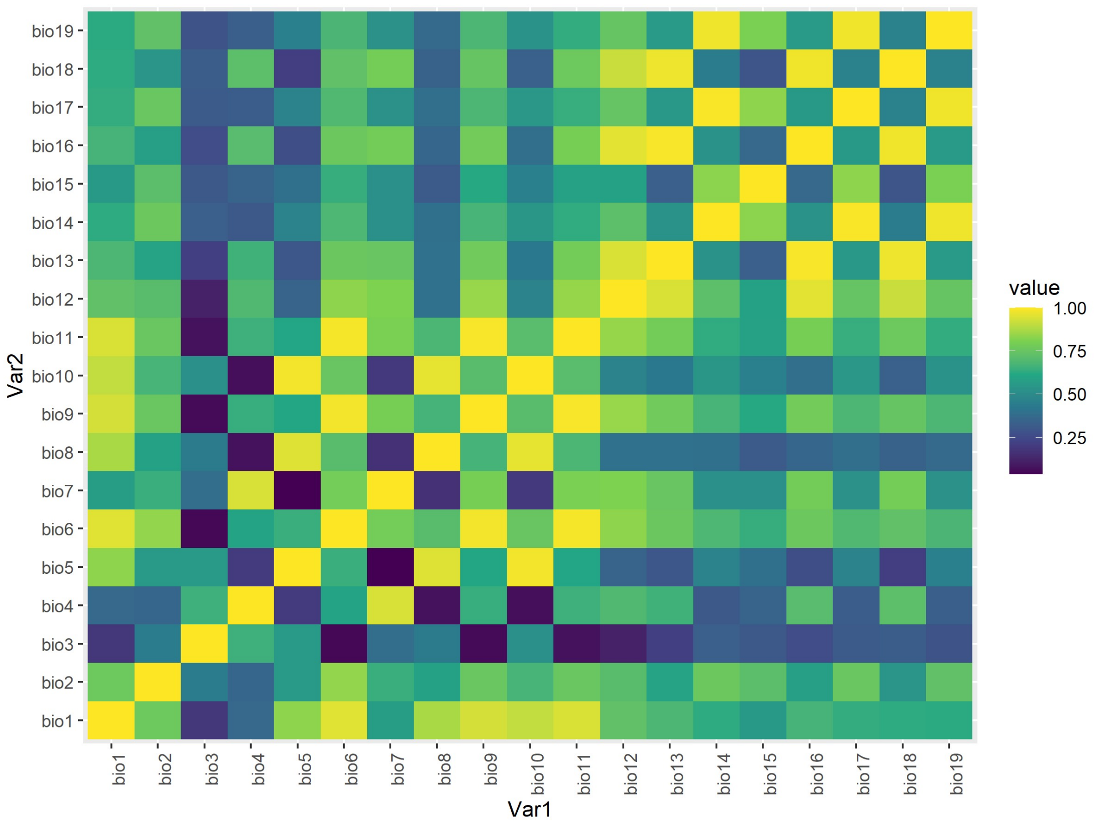

Figure S1 The heatmap of Pearson Correlation Matrix of 19 bioclimatic variables ($P <$ 0.05).

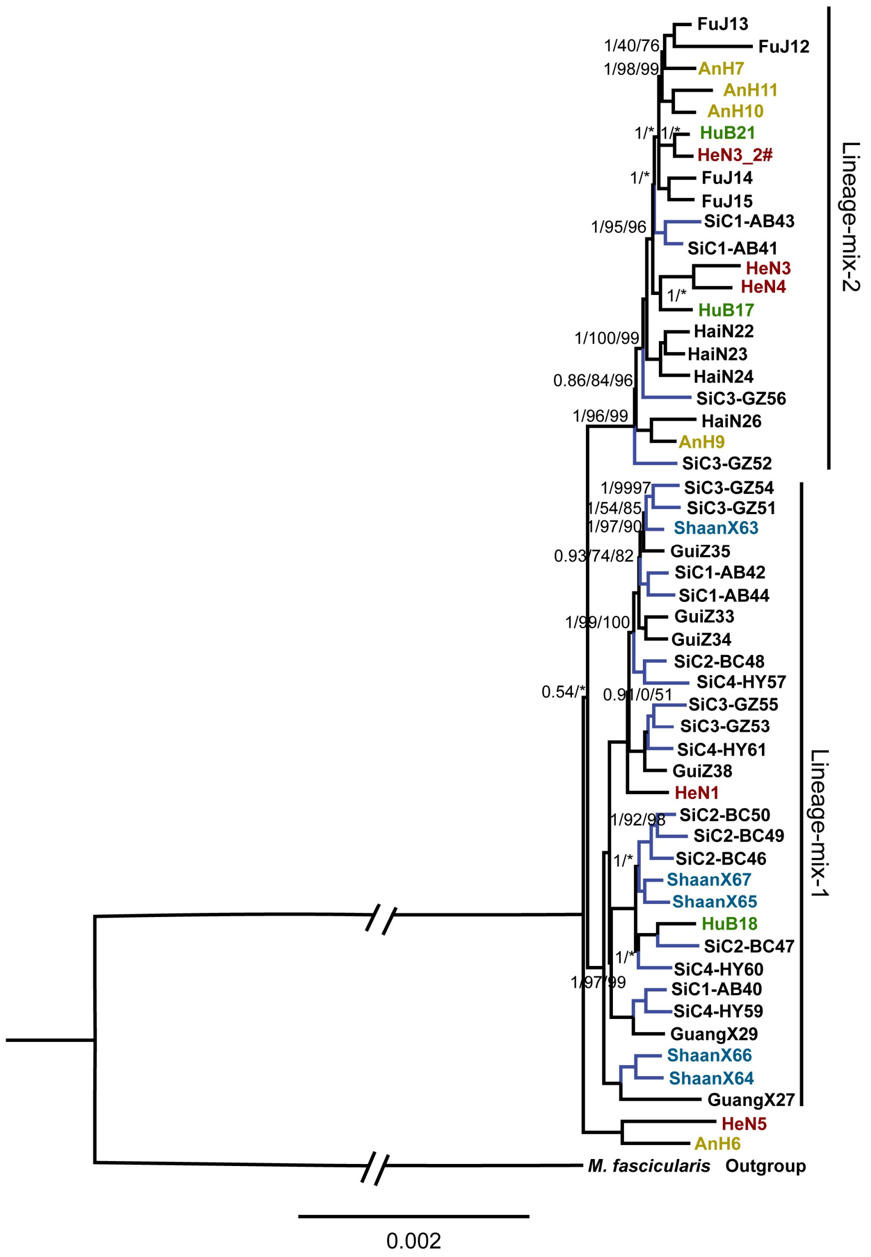

Figure S2 Bayesian inference (BI) and maximum likelihood (ML) phylogenetic trees from 52 male individuals based on Y-chromosome of Chinese rhesus macaques. Node support was presented above nodes and separated by slashes: Bayesian posterior probability/bootstrap support values for the maximum likelihood from MrBayes and IQ-tree analyses, respectively. The node support value was "1/100/100" no shown in tree. "*" indicated that the topology from BI analyses were incongruent with that from ML. Red "#" represented for our published study.

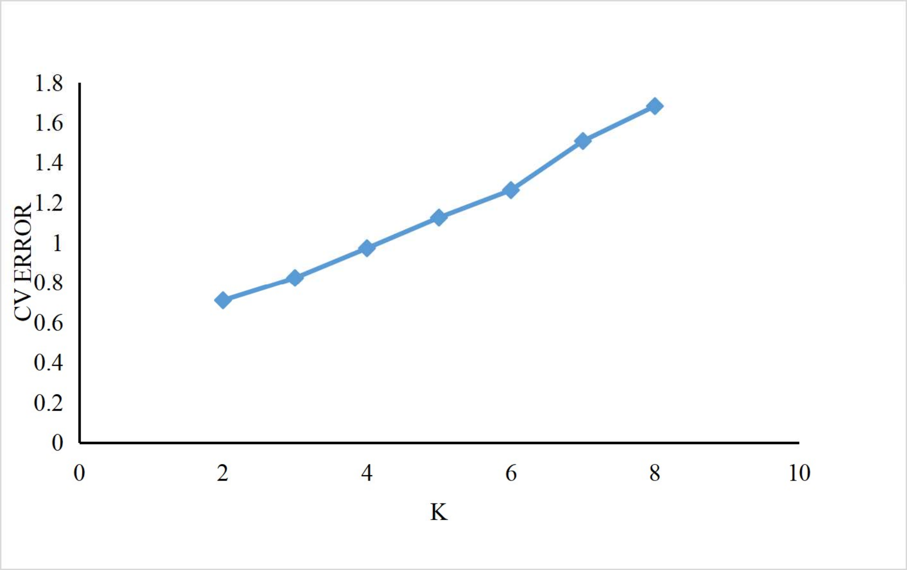

Figure S3 The cross-validation (CV) errors of ADMIXTURE runs with different clustering number (*K*) settings in rhesus macaques in subtropical and temperate China, show minimum value at *K* = 2 genetic groups.

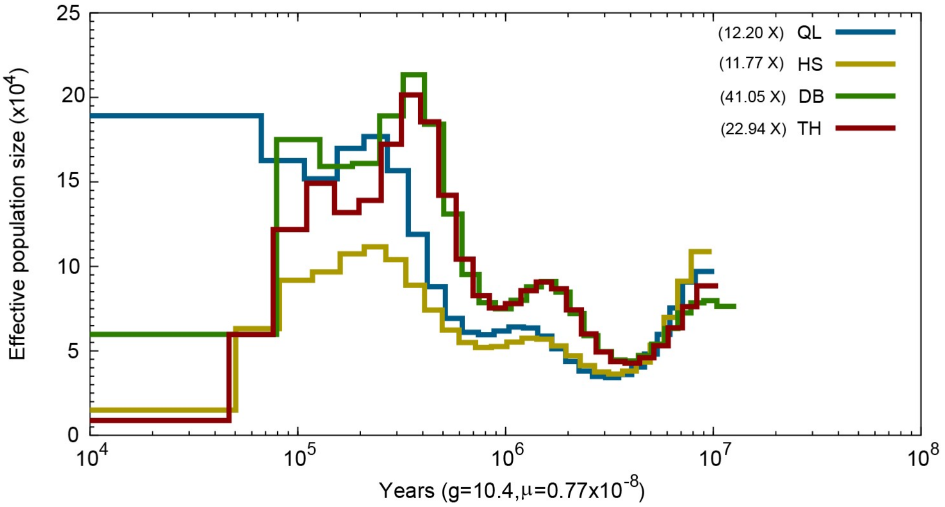

Figure S4 Historical changes in effective population size constructed using the PSMC applied on individual whole genome for rhesus macaques in subtropical and temperate China. The generation length (g) and the neutral mutation rate per site per generation (μ) were assumed to be 10.4 years and 0.77×10^{-8}, respectively. The ancient demographic history of these populations experienced a population reduction at the time of Xixiabangma Glaciation (XG, 1100-800 kya), followed by an expansion during the Mid-Pleistocene Inter-glaciation (800-200 kya), and the population size changes of Chinese rhesus macaque was similar to that estimated by Liu et al. (2018).

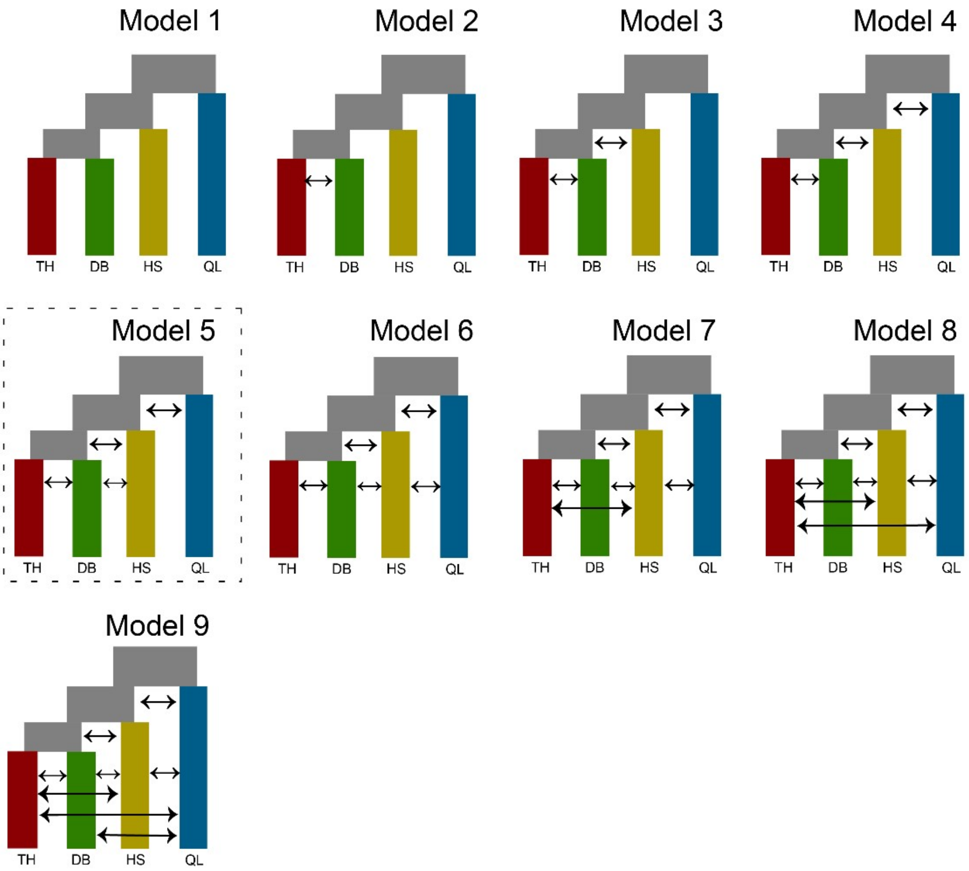

Figure S5 Models of different migration scenario between rhesus macaque populations in subtropical and temperate China and split time by G-PhoCS program.
